# Supplementary figures and images for: Tick-wildlife host-pathogen network interactions in Northern Africa
Source: PLoS One. 2025 Jul 15;20(7):e0327313. doi: 10.1371/journal.pone.0327313 (PMC12262905; doi:10.1371/journal.pone.0327313)

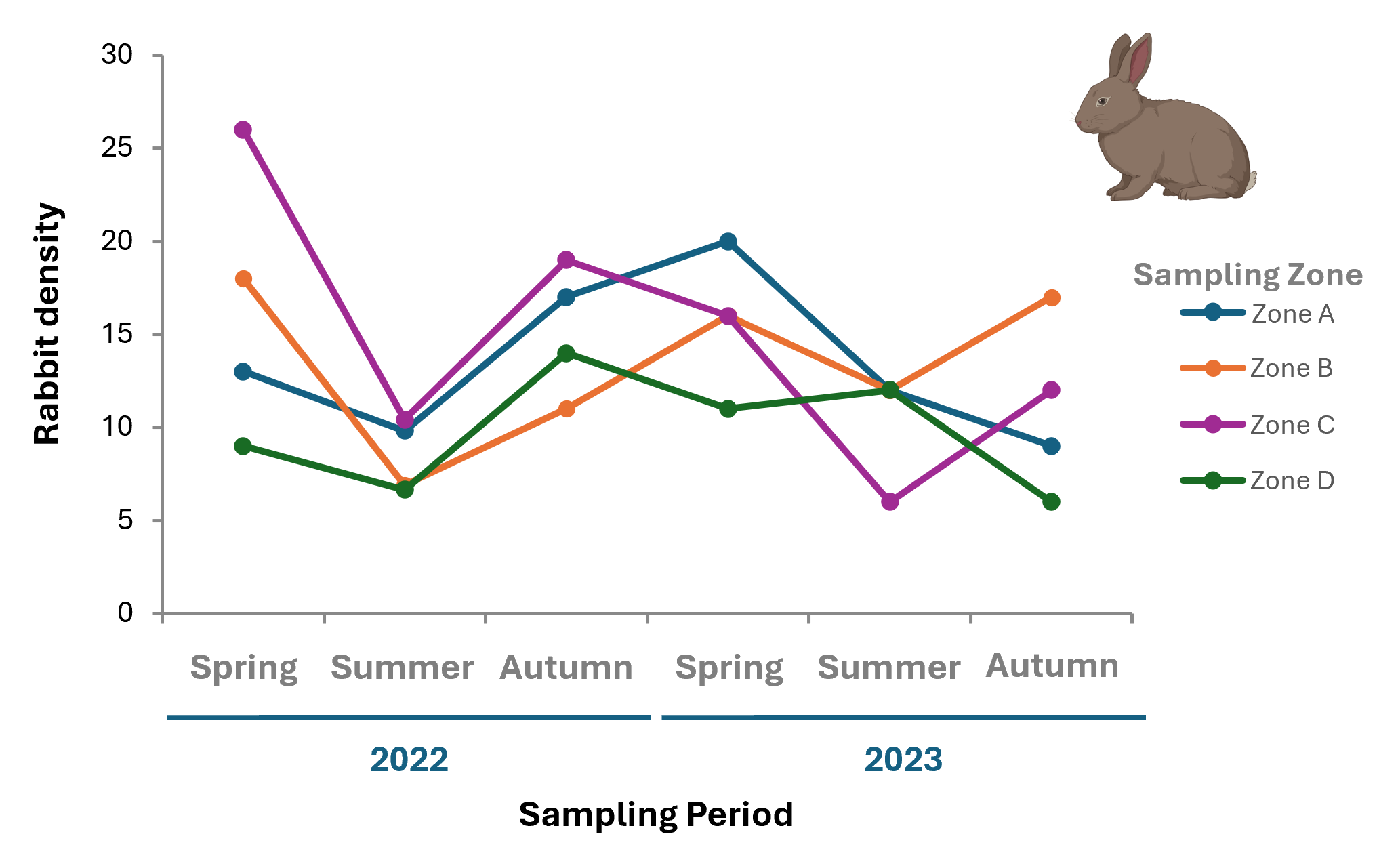

Supplement: S1 Fig — (TIF) [file pone.0327313.s001.tif]

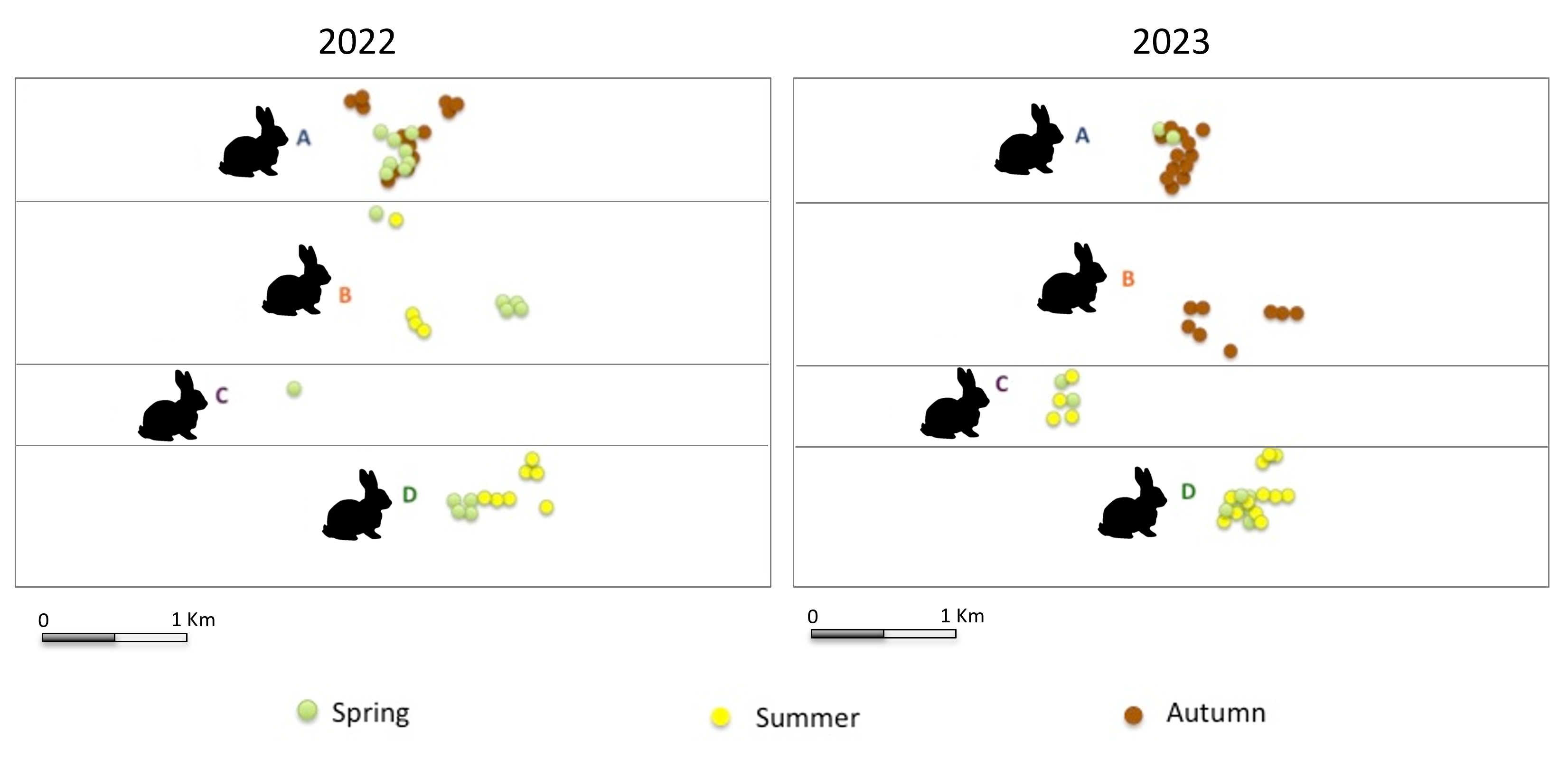

Supplement: S2 Fig — (TIF) [file pone.0327313.s002.tif]
