# Supplementary material for: Tick-wildlife host-pathogen network interactions in Northern Africa
Source: PLoS One. 2025 Jul 15;20(7):e0327313. doi: 10.1371/journal.pone.0327313 (PMC12262905; doi:10.1371/journal.pone.0327313)
Supplement: S2 Table — (DOCX) [file pone.0327313.s004.docx]

**Supporting information**

**S2 Table.** List of models organized by their Akaike Information Criteria (AIC).

| Models | AIC |
| --- | --- |
| Zone | 839 |
| Questing ticks | 830 |
| Rabbit density | 781 |
| Season | 508 |
| Season + rabbit density | 447 |
| Season + rabbit density + questing ticks | 426 |
| Season + rabbit density + questing ticks + zones | 401 |
