# Supplementary material for: Tick-wildlife host-pathogen network interactions in Northern Africa
Source: PLoS One. 2025 Jul 15;20(7):e0327313. doi: 10.1371/journal.pone.0327313 (PMC12262905; doi:10.1371/journal.pone.0327313)
Supplement: Attachment — Submitted filename: PONE-D-25-17487_reviewer_comments.pdf [file pone.0327313.s005.pdf]

PLOS ONE

Tick-wildlife host-pathogen network interactions in Northern Africa

--Manuscript Draft--

**Manuscript Number:**	PONE-D-25-17487
**Article Type:**	Research Article
**Full Title:**	Tick-wildlife host-pathogen network interactions in Northern Africa
**Short Title:**	Ticks network interactions in Africa
**Corresponding Author:**	Marta Simoes Correia Rafael<br>Instituto de Investigacion en Recursos Cinegeticos<br>Ciudad Real, SPAIN
**Keywords:**	Addax; Disease; Population dynamics; Of-host Tick; Vector-borne; Wild rabbit; Zoonoses
**Abstract:**	Ticks are hosts and vectors of zoonotic pathogens, posing a critical threat to public health and the conservation of animal host populations especially in North Africa. Tick-host-pathogen interactions are driven by tick spatial distribution and abundance, and the influence of biotic (animal hosts) and abiotic (environmental conditions) factors. The objectives of this study conducted in the Maamora Forest (Northwest Morocco) were (i) description of seasonal interactions network between off-host questing ticks and the wild hosts, rabbits (<i>Oryctolagus cuniculus</i>) and addax (<i>Addax nasomaculatus</i>), (ii) analysis of density-dependent and environmental effects in questing and on-rabbit ticks, and (iii) identification of tick-borne pathogens in questing and on-addax ticks. Results show questing and on-rabbit ticks (<i>Hyalomma lusitanicum</i>, <i>Rhipicephalus pusillus</i> and <i>H. aegyptium</i>) presented significant spatial and seasonal differences. Questing ticks were highly abundant in summer but infestation on rabbits was higher in spring. Spatially, areas with contact between rabbits and ungulates showed the highest tick infestations during summer. Ticks from rabbits were density-dependent and had a positive relationship with questing ticks. Addax was parasitized by <i>H. lusitanicum</i> ticks. Tick network interactions resulted in the presence of <i>Coxiella burnetii</i> in both questing and addax ticks (17-27%), and <i>Rickettsia aeschlimannii</i> in <i>H. lusitanicum</i> questing ticks (4%). These results support that ticks represent a challenge for human and animal health, as well as ecosystems in North Africa, emphasizing the need for long-term studies on their network interactions, seasonal activity patterns, and tick-borne pathogens in wildlife.
**Order of Authors:**	Marta Rafael
	Amalia Segura
	Rita Vaz-Rodrigues
	David Relimpio
	Oscar Rodríguez
	Gabriela de la Fuente
	Julio Isla
	Christian Gortázar
	José de la Fuente
**Additional Information:**	
**Question**	**Response**
**Financial Disclosure**<br><br>Enter a financial disclosure statement that describes the sources of funding for the	Yes

Powered by Editorial Manager® and ProduXion Manager® from Aries Systems Corporation

work included in this submission. Review the submission guidelines for detailed requirements. View published research articles from *PLOS ONE* for specific examples.<br><br>This statement is required for submission and **will appear in the published article** if the submission is accepted. Please make sure it is accurate.<br><br>**Funded studies**<br>Enter a statement with the following details:<br>• Initials of the authors who received each award<br>• Grant numbers awarded to each author<br>• The full name of each funder<br>• URL of each funder website<br>• Did the sponsors or funders play any role in the study design, data collection and analysis, decision to publish, or preparation of the manuscript?<br><br>Did you receive funding for this work?	
Please add funding details.<br>as follow-up to "**Financial Disclosure**<br><br>Enter a financial disclosure statement that describes the sources of funding for the work included in this submission. Review the submission guidelines for detailed requirements. View published research articles from *PLOS ONE* for specific examples.<br><br>This statement is required for submission and **will appear in the published article** if the submission is accepted. Please make sure it is accurate.	This research was partly supported by the 2022-GRIN-34227 grant, funded by the University of Castile-La Mancha (UCLM), Spain and EU-FEDER. R. Vaz-Rodrigues was supported by a doctoral contract (2022-PRED-20675), from UCLM and co-financed by the European Social Fund (ESF).

**Funded studies**<br>Enter a statement with the following details:<br>• Initials of the authors who received each award<br>• Grant numbers awarded to each author<br>• The full name of each funder<br>• URL of each funder website<br>• Did the sponsors or funders play any role in the study design, data collection and analysis, decision to publish, or preparation of the manuscript?<br><br>Did you receive funding for this work?"	
Please select the country of your main research funder (please select carefully as in some cases this is used in fee calculation).<br>as follow-up to "**Financial Disclosure**<br><br>Enter a financial disclosure statement that describes the sources of funding for the work included in this submission. Review the submission guidelines for detailed requirements. View published research articles from *PLOS ONE* for specific examples.<br><br>This statement is required for submission and **will appear in the published article** if the submission is accepted. Please make sure it is accurate.<br><br>**Funded studies**<br>Enter a statement with the following details:<br>• Initials of the authors who received each award<br>• Grant numbers awarded to each author<br>• The full name of each funder<br>• URL of each funder website<br>• Did the sponsors or funders play any role in the study design, data collection and analysis, decision to publish, or preparation of the manuscript?<br><br>Did you receive funding for this work?"	SPAIN - ES
**Competing Interests**	The authors have declared that no competing interests exist.

Use the instructions below to enter a competing interest statement for this submission. On behalf of all authors, disclose any competing interests that could be perceived to bias this work—acknowledging all financial support and any other relevant financial or non-financial competing interests.<br><br>This statement is required for submission and **will appear in the published article** if the submission is accepted. Please make sure it is accurate and that any funding sources listed in your Funding Information later in the submission form are also declared in your Financial Disclosure statement.<br><br>View published research articles from *PLOS ONE* for specific examples.<br><br>**NO authors have competing interests**<br><br>Enter: *The authors have declared that no competing interests exist.*<br><br>**Authors with competing interests**<br><br>Enter competing interest details beginning with this statement:<br><br>*I have read the journal's policy and the authors of this manuscript have the following competing interests: [insert competing interests here]*<br><br>* typeset	
**Ethics Statement**<br><br>Enter an ethics statement for this submission. This statement is required if the study involved:<br><br>• Human participants<br>• Human specimens or tissue<br>• Vertebrate animals or cephalopods<br>• Vertebrate embryos or tissues	N/A

Powered by Editorial Manager® and ProduXion Manager® from Aries Systems Corporation

• Field research<br><br>Write "N/A" if the submission does not require an ethics statement.<br><br>General guidance is provided below. Consult the submission guidelines for detailed instructions. **Make sure that all information entered here is included in the Methods section of the manuscript.**	

Powered by Editorial Manager® and ProduXion Manager® from Aries Systems Corporation

**Format for specific study types**<br><br>**Human Subject Research (involving human participants and/or tissue)**<br>• Give the name of the institutional review board or ethics committee that approved the study<br>• Include the approval number and/or a statement indicating approval of this research<br>• Indicate the form of consent obtained (written/oral) or the reason that consent was not obtained (e.g. the data were analyzed anonymously)<br><br>**Animal Research (involving vertebrate animals, embryos or tissues)**<br>• Provide the name of the Institutional Animal Care and Use Committee (IACUC) or other relevant ethics board that reviewed the study protocol, and indicate whether they approved this research or granted a formal waiver of ethical approval<br>• Include an approval number if one was obtained<br>• If the study involved *non-human primates*, add *additional details* about animal welfare and steps taken to ameliorate suffering<br>• If anesthesia, euthanasia, or any kind of animal sacrifice is part of the study, include briefly which substances and/or methods were applied<br><br>**Field Research**<br><br>Include the following details if this study involves the collection of plant, animal, or other materials from a natural setting:<br>• Field permit number<br>• Name of the institution or relevant body that granted permission	
**Data Availability**<br><br>Authors are required to make all data underlying the findings described fully available, without restriction, and from the time of publication. PLOS allows rare exceptions to address legal and ethical concerns. See the PLOS Data Policy and FAQ for detailed information.	Yes - all data are fully available without restriction

A Data Availability Statement describing where the data can be found is required at submission. Your answers to this question constitute the Data Availability Statement and **will be published in the article**, if accepted.<br><br>**Important:** Stating 'data available on request from the author' is not sufficient. If your data are only available upon request, select 'No' for the first question and explain your exceptional situation in the text box.<br><br>Do the authors confirm that all data underlying the findings described in their manuscript are fully available without restriction?	
**Describe where the data may be found in full sentences. If you are copying our sample text, replace any instances of XXX with the appropriate details.**<br><br>• If the data are **held or will be held in a public repository**, include URLs, accession numbers or DOIs. If this information will only be available after acceptance, indicate this by ticking the box below. For example: *All XXX files are available from the XXX database (accession number(s) XXX, XXX.).*<br>• If the data are all contained **within the manuscript and/or Supporting Information files**, enter the following: *All relevant data are within the manuscript and its Supporting Information files.*<br>• If neither of these applies but you are able to provide **details of access elsewhere**, with or without limitations, please do so. For example:<br><br>*Data cannot be shared publicly because of [XXX]. Data are available from the XXX Institutional Data Access / Ethics Committee (contact via XXX) for researchers who meet the criteria for access to confidential data.*<br><br>*The data underlying the results presented in the study are available from (include the name of the third party*	All relevant data are within the manuscript and its Supporting Information files.

and contact information or URL).<br>• This text is appropriate if the data are owned by a third party and authors do not have permission to share the data.<br><br>* typeset	
Additional data availability information:	

Powered by Editorial Manager® and ProduXion Manager® from Aries Systems Corporation

Manuscript Click here to access/download;Manuscript;Manuscript.docx

Tick-wildlife host-pathogen network interactions in Northern Africa

Marta Rafael[1,&*], Amalia Segura[2&], Rita Vaz-Rodrigues[1], David Relimpio[1], Oscar Rodríguez[2], Gabriela de la Fuente[3], Julio Isla[3], Christian Gortázar[1], José de la Fuente[1,4*].

[1] SaBio. Instituto de Investigación en Recursos Cinegéticos IREC-CSIC-UCLM-JCCM, Ronda de Toledo 12, 13005 Ciudad Real, Spain.

[2] BP 30, Sidi Allal el Bahraoui 15250, Morocco.

[3] Sabiotec, Edificio incubadora de empresas UCLM, Camino de Moledores s/n, Ciudad Real 13071, Spain.

[4] Department of Veterinary Pathobiology, Center for Veterinary Health Sciences, Oklahoma State University, Stillwater, OK 74078, USA.

* Corresponding autors

E-mail: marta.srafael@uclm.es (MR); jose_delafuente@yahoo.com (JF)

& These authors contributed equally to this work

Emails & ORCID IDs

Marta Rafael: marta.srafael@uclm.es; 0000-0001-5923-984X

Amalia Segura: amaliasegura@gmail.com; 0000-0002-0319-829X

Rita Vaz-Rodrigues: rita.vrodrigues@uclm.es; 0000-0002-0411-7738

David Relimpio: david.relimpio@uclm.es; 0009-0005-7443-8667

Oscar Rodríguez: oscar.rodriguez.vet@gmail.com

Gabriela de la Fuente: gabriela@sabiotec.es

Julio Isla: julio.isla@sabiote.es; 0000-0002-7975-829X

Christian Gortázar: christian.gortazar@uclm.es; 0000-0003-0012-4006

José de la Fuente: jose_delafuente@yahoo.com; 0000-0001-7383-9649

Abstract

Ticks are hosts and vectors of zoonotic pathogens, posing a critical threat to public health and the conservation of animal host populations especially in North Africa. Tick-host-pathogen interactions are driven by tick spatial distribution and abundance, and the influence of biotic (animal hosts) and abiotic (environmental conditions) factors. The objectives of this study conducted in the Maamora Forest (Northwest Morocco) were (i) description of seasonal interactions network between off-host questing ticks and the wild hosts, rabbits (*Oryctolagus cuniculus*) and addax (*Addax nasomaculatus*), (ii) analysis of density-dependent and environmental effects in questing and on-rabbit ticks, and (iii) identification of tick-borne pathogens in questing and on-addax ticks. Results show questing and on-rabbit ticks (*Hyalomma lusitanicum, Rhipicephalus pusillus* and *H. aegyptium*) presented significant spatial and seasonal differences. Questing ticks were highly abundant in summer but infestation on rabbits was higher in spring. Spatially, areas with contact between rabbits and ungulates showed the highest tick infestations during summer. Ticks from rabbits were density-dependent and had a positive relationship with questing ticks. Addax was parasitized by *H. lusitanicum* ticks. Tick network interactions resulted in the presence of *Coxiella burnetii* in both questing and addax ticks (17-27%), and *Rickettsia aeschlimannii* in *H. lusitanicum* questing ticks (4%). These results support that ticks represent a challenge for human and animal health, as well as ecosystems in North Africa, emphasizing the need for long-term studies on their network interactions, seasonal activity patterns, and tick-borne pathogens in wildlife.

Keywords: Addax; Disease; Population dynamics; Of-host Tick; Vector-borne; Wild rabbit; Zoonoses

Introduction

Ticks are important ectoparasites as vectors and reservoirs of pathogenic bacterial, viral, and protozoan microorganisms [1]. As a result, intricate eco-epidemiological webs for pathogen transmission can emerge, potentially impacting both animal and human health. Unlike other vector-borne parasites, ticks present a distinctive life cycle involving one or multiple hosts throughout their lifespan. Depending on whether the tick life cycle is of one, two or three hosts, they pass through various host species. Immature stages, such as larva and nymphs, tend to parasitise animals of a smaller size, like lagomorphs, rodents, or lizards, while adult stages parasitise large to medium-sized animals, for instance, camels and ungulates [2]. In between life stages, ticks molt into the next stage either on the first host species (one-host cycle) or in the environment (two or three-host cycle). Hence, between each life cycle, off-host ticks can be identified questing, a host-seeking behaviour where hard ticks ascend vegetation and wait for a passing host to attach [3]. Ticks' abundance and spatial distribution are highly dependent on biotic factors, including host population, individual host characteristics and the abundance of potential hosts, typically gathering in environments with higher host aggregation [4,5], as well as abiotic environmental variables such as temperature and seasonality [6,7]. In North Africa, ticks share a Mediterranean climate with four seasons, characterized by hot and dry summers and wet winters, supporting a rich diversity of tick species and tick-borne pathogens [8]. Numerous wildlife species have been recorded as hosts, potentially transporting ticks and pathogens into and out of North Africa through migratory birds or traded animals (livestock and exotic pets). For instance, *Hyalomma aegyptium* ticks has been recorded on *Testudo graeca* spur-thighed tortoises (considered as an exotic pet) in Morocco, Algeria and Turkey [9–11]. In mammals, *H. dromedarii, H. marginatum* or *H. dentritum* have been found on addax in Morocco and Tunisia [12–14]. Moreover, *H.*

excavatum and *H. dromedarii* adult ticks have been reported on scimitar-horned oryx in Tunisia [13]. Nevertheless, little is known about the role of certain wild species as reservoirs, such as small mammals like the wild rabbit *Oryctolagus cuniculus*, which has been documented in Egypt and other Mediterranean environments as a carrier of *Coxiella burnetii* associated to *H. lusitanicum* [15,16]. Furthermore, wild rabbits, as highly abundant populations and prolific breeders, may pose a higher risk of transmitting the infection [17]. As the number of susceptible hosts for the same tick species increases, the transmission of numerous infectious agents across different species is facilitated, establishing intricate networks for pathogen transmission, leading to significant epidemiological implications [18]. Indeed, ticks from North Africa have been associated with several infectious agents, such as *Anaplasma phagocytophylum*, *Babesia bovis*, *B. caballi* and *B. divergens*, *Borrelia lusitaniae* and *B. burgdorferi*, the Crimean-Congo haemorrhagic fever virus (CCHFV), *C. burnetii*, *Ehrlichia ewingii*, *Rickettsia aeschlimannii*, *R. africae* and *R. massiliae*, *Theileria annulate*, among others [19,2,20]. Furthermore, in North African countries ticks have also been previously reported in humans, such as *Ixodes ricinus*, *H. aegyptium*, *H. marginatum*, *Rhipicephalus bursa* or *R. sanguineus*, and present a zoonotic potential for anaplasmosis, borreliosis, ehrlichiosis, Q fever, rickettsiosis, babesiosis or CCHFV [21,22,20,5].

In this study, we investigate the network interactions between off-host ticks (present in vegetation searching for a host, hereafter questing ticks), wild rabbits as intermediate hosts and ungulates in Maamora forest (Northwest Morocco), where livestock is abundant [23]. The Saharan antelope addax (*Addax nasomaculatus*), a “critically endangered” ungulate [24], has been identified as a carrier for several tick-borne pathogens, including *Rickettsia* spp., *Ehrlichia* spp., *Anaplasma* spp. and *C. burnetti* [14], with the latter also shared by other wild ungulates (33-62% prevalence, unpublished data). Hence, this

species was selected as the ungulate animal model to study tick life cycle transmission, aiming to increase the knowledge of potential tick-borne pathogens affecting this species (e.g. plausible *C. burnetii* causing abortion). Particularly, this study intends to i) determine the abundance and diversity of questing ticks and ticks collected from rabbits seasonally, ii) determine density-dependent and environmental effects in questing ticks and ticks from wild rabbits and iii) determine tick-borne pathogens circulating in questing ticks and those infecting addax. The results will shed light on the network of ticks and tick-borne infectious agents, potentially emphasizing the need for a One-Health approach to prevent the transmission of zoonotic diseases in trans-Mediterranean environments affecting both Europe and North Africa [20].

Materials and methods

Study site

The study was conducted within the Maamora Forest (Northwest Morocco), which is characterised by a semi-arid to sub-humid climate. The annual precipitation ranges from 300–500 mm, mostly centred in autumn and winter and dry periods from June to September. In recent years main rainfall has been observed in winter and spring, representing 55% and 71% of annual rainfall in 2022 and 2023, respectively [25]. A significant increase in temperature has been observed, especially during spring and summer months, increasing on average by more than 4% per decade [26]. The Maamora forest is mainly covered by cork oak trees (*Quercus suber*), endemic wild peer (*Pyrus mamorensis*) with tall scrubs of wild olive (*Olea europaea*), green olive (*Phillyrea latifolia*) and mastic (*Pistacia lentiscus).* There are high densities of livestock, particularly cows and sheep [23].

The study took place on a private protected reserve (3000 ha), characterised by a varied and rich undergrowth (i.e. high species richness and cover), evidenced by its variety of species (Mediterranean brooms [*Genista linifolia, Cytisus arboreus*, *Stauracanthus genistoides*], dwarf palm [*Chamaerops humilis*], French lavender [*Lavandula stoechas*], sparrow-wort [*Tymelea lytroides*] and sage-leave rockrose [*Cistus salviifolius*]) and surface coverage, compared to other unprotected areas in Maamora which are highly overgrazed by livestock [27]. The undergrowth and mammal density varies in the reserve. Therefore, our study was done in four zones 5 km apart each (A, B, C and D; Fig 1), with similar scrub diversity but with varying scrub cover as follows, over 50% in B and C, over 40% in A and over 25% in D and bare ground absent in B and C and over 15% in A and D. While there are rabbits in the four study zones, in zones B and D there are no ungulates due to fences separating them. The density of addax in A and B are 25 and 44 individuals (in 800 ha each), respectively. Apart from addax, there are other tick hosts present in the study area, such as spur-thighed tortoises, ~~have~~ been documented to present high tick infestation intensity [mean of 4 *H. aegyptium* tick/tortoise; [9,11]], and there are other plausible hosts like hedgehogs.

Fig 1. Study area. Representation of the study area, divided into zones A, B, C, and D, and abundance of vegetation (scrub), rabbits and addax across the study area. Rabbits' abundance and scrub cover vary throughout the study area, and there are no ungulates in zones B and D. The map was generated using the ArcGIS software, version 10.8.

Tick sampling and rabbit density

Questing ticks are host-seeking on top of a plant (scrub or herb) for a chance to attach to a passing host, such as tortoises, hedgehogs (*Atelerix algirus*), rodents, etc. Questing ticks were collected in the four study zones and in four sampling periods, June 2022, March, June, and November 2023, corresponding to spring, summer and autumn seasons. The flagging method, used to collect ticks from vegetation, consisted of a cloth towel strapped

to a long wooden edge and dragged behind the operator by a rope attached to the wooden log [2]. Three individuals conducted the sampling by dragging the cloth across ground-level vegetation, with a sampling effort of 1 hour per study zone. Ticks were placed into plastic vials containing RNAlater (Thermo Fisher, Waltham, MA, USA) and stored at -20 ºC until further analysis.

Ticks infesting rabbits were collected from five randomly selected dead individuals in each sampling zone in the same periods as questing ticks. Dead rabbits were the result of traffic crashes and naturally dead animals through transects coinciding with rabbit surveys. In addition, coinciding with study periods and zones, rabbit populations were surveyed and data was georeferenced for determining densities and spatial distribution. Rabbit populations were surveyed at night, using spotlights while driving at speeds of 20-30 km/h through human tracks using spotlights along 4 transects of 3-5 km length and 30 m wide in non-brightest nights (full moon). Hotspot analysis for determining aggregation patterns was accomplished by GIS (ArcGIS 10.8).

Ticks infesting addax, as a potential ungulate model for tick infestation, were collected from ten live animals during the annual sanitary control in November 2022. The aim was to determine their role in the network interaction of ticks and to explore vegetation and host-associated tick-borne pathogens. Ticks were placed into plastic vials containing RNAlater (Thermo Fisher, Waltham, MA, USA) and stored at -20 ºC until further analysis.

DNA/RNA isolation and PCR for pathogen detection in questing and addax ticks

A representative subsample of collected ticks was used for DNA extraction and analysis of infectious agents (Table S1). Ticks were morphologically ~~classified according to~~ [2]. Nucleic acid extraction was accomplished from individual and tick pools (mean of 3 ticks/pool). ~~Pools were designed~~ randomly, ranging from 1 to 6 ticks/pool. DNA and

RNA were extracted from the internal tissues of ticks, discarding the external cuticle, using 250 μL of TRI Reagent (Sigma-Aldrich, St. Louis, USA), and following the manufacturer's instructions. The concentration (ng/μL) and purity of samples were evaluated using a Nanodrop One spectrophotometer (Thermo Scientific, Waltham, USA), through the quantification of the nucleic acids at an optical density of 260 nm (OD_{260}), and the ratio of absorbance at 260/280 nm. The quality of the extraction protocol and morphological identification of tick species was confirmed by DNA barcoding of the *mitochondrial 16S ribosomal DNA* (*16S rDNA*) gene and the *cytochrome oxidase subunit I* (*COI*) gene (Table 1). Ticks used for molecular identification included four individual adult ticks collected from vegetation, four individual adult ticks, and three pools of nymphs, constituted by two to four nymphs collected from rabbits.

A random sample of addax's ticks (n = 49 ticks, 11 tick pools) and ticks from vegetation (n = 84 ticks, 24 pooled ticks) were tested using conventional polymerase chain reaction (PCR) aimed at detecting the presence of pathogenic agents, such as *Anaplasma* spp., *Babesia* spp., *C. burnetii*, *Rickettsia* spp., *Theileria* spp. and a nested reverse transcription (RT)-PCR for the identification of the CCHFV. Table 1 provides information on the specific targeted regions for each PCR assay, the protocol, and the specific primers used.

Table 1. Primers and PCR conditions. List of primers and PCR protocol according to the pathogenic agent analysed.

Pathogen and/or target gene	Sequence 5'-3' (F: Forward / R: Reverse)	Fragment (base pairs)	Annealing (ºC)	Reference
16S rDNA	F: CCGGTCTGAACTCAGATCAAGT<br>R: CTGCTCAATGATTTTTTAAATTGCTGTGG	460 bp	48	[14]
COI	F: GGTCAACAAATCATAAAGATATTGG<br>R: TAAACTTCAGGGTGACCAAAAAATCA	650 bp	50	[28]

Pathogen and/or target gene	Sequence 5’-3’ (F: Forward / R: Reverse)	Fragment (base pairs)	Annealing (ºC)	Reference
***Anaplasma* spp. (*16S rRNA*)**	F: CAGAGTTTGATCCTGGCTCAGAACG<br>R: GAGTTTGCCGGGACTTCTTCTGTA	421 bp	42	[29]
***Anaplasma* spp. (*msp5*)**	F: GCATAGCCTCCGCGTCTTTC<br>R: TCCTCGCCTTGGCCCTCAGA	456 bp	54	[29]
***Anaplasma* spp. (*msp4*)**	F: CGGATCCTTAGCTGAACAGGAATCTTGC<br>R: GGGAGCTCCTATGAATTACAGAGAATTG TTTAC	849 bp	60	[29]
***Coxiella burnetii* (*IS111a*)**	F: CAAGAATGATCGTAACGATGCGC<br>R: CTCGTAACACCAATCGCTTCG	349	63	[30]
**Crimean-Congo Haemorrhagic Fever vírus (S segment)**	F1: TTGTGTTCCAGATGGCCAGC<br>R1: CTTAAGGCTGCCGTGTTTGC<br>F2: GAAGCAACCAARTTCTGTGC<br>R2: AAACCTATGTCCTTCCTCC	211	60 and 57	[31]
***Rickettsia* spp. (*16S rRNA*)**	F: AGAGTTTGATCCTGGCTCAG<br>R: AACGTCATTATCTTCCTTGC	416	54	[14]
***Rickettsia* spp. (*ompA*)**	F: ATGGCGAATATTTCTCCAAAA<br>R: AGTGCAGCATTCGCTCCCCCT	630	54	[32]
***Rickettsia* spp. (*ompB*)**	F: GGGTGCTGCTACACAGCAGAA<br>R: CCGTCACCGATATTAATTGCC	618	53	[33]
***Rickettsia* spp. (*gltA*)**	F: GGGGGCCTGCTCACGGCGG<br>R: ATTGCAAAAAGTACAGTGAACA	360	45	[34]
***Rickettsia* spp. (*recA*)**	F: TGCTTTTATTGATGCCGAGC<br>R: CTTTAATGGAGCCGATTCTTC	428	52	[33]
***Rickettsia* spp. (*atpA*)**	F: ACATATCGAGATGAAGGCTCC<br>R: CCGAAATACCGACATTAACG	731	48	[33]
***Babesia* / *Theileria* spp. (18S rRNA)**	F: GTCTTGTAATTGGAATGATGG<br>R: CCAAAGACTTTGATTTCTCTC	550	55	[35]

The PCR reactions were performed in a 25 μL volume, including 12.5 μL of PCR Master Mix 2x (Promega, Madison, WI, USA), 1 μL of each primer (10 μM working solution), 9 μL of RNase-free water (Thermo Scientific), and 1.5 μL of DNA sample. For the nested RT-PCR assessment of CCHFV, the commercial kit Access RT-PCR System (Promega, Fitchburg, WI, USA) was used according to the manufacturer's instructions. The PCRs were conducted in a C1000 touch PCR thermal cycler (Bio-Rad, Hercules, CA, USA), with the specific PCR fragments visualized in a 1.5% agarose gel stained with GelRed (Biotium, Fremont, CA, USA) under UV transillumination.

Phylogenetic analysis

Suspected PCR-positive samples were purified and sequenced using the Sanger method at Secugen (Madrid, Spain). Sequences were assessed in Chromas software (version 2.6.6.), and homology analysis was conducted using the National Center for Biotechnology Information (NCBI) database, employing the Basic Local Alignment Search Tool (BLAST). The obtained new sequences were deposited in GenBank database. Multiple sequence alignment was carried out between obtained sequences and reference sequences from GenBank using the Multiple Sequence Comparison by Log-Expectation (MUSCLE) algorithm. Phylogenetic analysis was performed in MEGA software (version 11.0.13) to evaluate the genetic association between retrieved sequences and reference samples. The best-fit model was selected based on the corrected Akaike Information Criterion (cAIC), and phylogenetic trees for positive infectious agents were generated using Maximum Likelihood and Neighbor-Joining methods. To ensure the reliability of produced trees, 1000 bootstrap replicates were implemented.

Statistical analysis

The prevalence of questing ticks and rabbit ticks were calculated to analyse differences among seasons and study zones by Pearson's Chi-squared test (χ^2) and Fisher's exact test, respectively, both followed by a post-hoc test (Bonferroni adjustment). The relationship between the response variable rabbit ticks and predictors rabbit population density, season, spatial distribution (zones) and questing ticks was performed using a generalized linear model (GLM) with a Poisson distribution and logarithmic link function. The most parsimonious model was then selected using a forward stepwise procedure based on Akaike Information Criteria (AIC) [36]. Statistical analyses were performed with R software, version 4.2.3 [37].

Results

Questing ticks, rabbit ticks and rabbit densities

Questing ticks (n = 3754) were mostly adults (1966 females and 1787 males) and one nymph. They were morphologically identified as *H. lusitanicum* (n = 3653), *H. aegyptium* (n = 98), and *Rhipicephalus* spp. (n = 3).

Seasonally, higher numbers of questing ticks were collected in summer 2023 and 2022 (1520 and 1229, respectively), when compared with spring (n = 883) and autumn (n = 121) (Fig 2A). Annual summer differences coincide with high and low previous spring rainfall (139 and 56 mm, in 2022 and 2023, respectively) and high temperature (36ºC) (Tiflet Meteorological Station). Spatially, more questing ticks were collected in zones C and A (1948 and 1127, respectively) than in B and D (631 and 47, respectively).

Our results ~~evidence~~ significant differences in the number of questing ticks collected throughout the different study zones and seasons (χ^2= 224.68, *p*-value <0.05; Fig 2B). Fig 2B illustrates the percentage of ticks collected across sampling zones and seasons and highlights the significant relations between collected and expected ticks in spring 2023 in zones A and C, summer 2023 in zone B, and autumn 2023 in zone D.

Fig 2. Questing ticks. (A) Seasonal and spatial distribution of collected questing ticks across zones A, B, C, and D and seasons. (B) Percentage of questing ticks (size of dots), representation of residuals and adjusted *p*-values from Pearson's Chi-Squared Test post-hoc analysis. Residuals indicate higher (green) or lower (red) values than expected, corresponding to statistically significant correlations (*p*-values < 0.05). Non-statistical results are shown with a grey dot.

Rabbit ticks (n = 1137) were mainly adults (387 males and 200 females), nymphs (n = 474) and larvae (n = 76). Mean infestation rate of 18 ± 9.43 ticks/rabbit. Ticks were morphologically classified as *H. lusitanicum* (4 adults), *Hyalomma* spp. (347 nymphs and larvae) and *Rhipicephalus pusillus* (583 adults, 203 nymphs, and larvae).

Seasonally, more ticks were collected in spring 2023 (n = 417) and summer 2023 and 2022 (n = 391 and 272, respectively), compared with autumn (n = 57) (Fig 3A). Spatially, a larger number of ticks were collected in zone B (n = 363), compared with zones A, D and C (n = 275, 262 and 237, respectively).

Our results show significant differences in the number of ticks collected from rabbits throughout the different study zones and seasons (χ^2= 388.32, *p*-value < 0.05, Fig 3B). Rabbit ticks were significantly higher in summer 2022 for zones B and C, in spring 2023 for zone D, in summer 2023 for zone A, and in autumn 2023 for zone D (Fig 3B).

Fig 3. Wild rabbit (*Oryctolagus cuniculus*) (A) Seasonal and spatial distribution of collected rabbits' ticks across zones A, B, C, and D and seasons. (B) Percentage of ticks collected from wild rabbits (size of dots), representation of residuals and adjusted p-values from Fisher's exact test post-hoc analysis. Residuals indicate higher (green) or

lower (red) values than expected, corresponding to statistically significant correlations (p-values < 0.05). Non-statistical results are shown with a grey dot.

Rabbit densities varied from 7 to 26 individuals/km^2 in the four zones and seasons, with zone D presenting slightly lower densities compared with the other zones. Spring, followed by autumn, shows the highest densities (Fig S1). Rabbits were aggregated in different zones according to season and year. In both summers, the aggregation was observed in zones C and D, in spring in zones A, C and D and in autumn in zones A and B (Fig S2).

Model analysis revealed that tick parasitisation in rabbits was significantly related to rabbit densities, season, zone and questing ticks (Table 2). Higher density of rabbits and questing ticks increases rabbit parasitisation.

Table 2. Statistical parameters of the generalised linear model (GLM) used to explain rabbit ticks according to rabbit density, questing ticks, season and spatial distribution (zone). See Table S2 for model selection.

	**Estimate**	**Std error**	**Z value**	**p-value**
**Rabbit density**	0.077	0.009	8.854	<0.01
**Questing ticks**	0.002	0.001	3.093	<0.01
**Spring23**	1.505	0.154	9.741	<0.01
**Summer22**	0.855	0.179	4.756	<0.01
**Summer23**	1.579	0.188	8.374	<0.01
**Zone B**	0.501	0.104	4.787	<0.01
**Zone C**	-0.555	0.147	-3.764	<0.01
**Zone D**	0.749	0.178	4.196	<0.01

Tick molecular identification and phylogeny

The morphological identification of ticks was confirmed by barcoding analysis of individual ticks collected from vegetation (questing) and attached to wild rabbits, corroborating its previous classification. Questing ticks (Isolate numbers 8, 12 and 15) were molecularly identified as *H. lusitanicum*. BLAST analysis revealed a sequence sharing of 99.78 – 99.80% nucleotide identity with the *16S rDNA* and/or *COI* genes of *H. lusitanicum* ticks attached to rabbits (GenBank accession number MZ420716) or unattached in vegetation (GenBank accession number EU827708). Adult ticks collected from rabbits (Isolate numbers 34, 38, 39 and 40) were molecularly identified as *R. pusillus* with a 99.47 – 100% nucleotide identity to the *16S rDNA* gene (GenBank accession numbers Z97883 and OL454849). On the other hand, nymphs (Isolate numbers 29 and 42) shared a 99.34 – 100% nucleotide identity with the *H. lusitanicum 16S rDNA* and/or *COI* genes (GenBank accession numbers OR091373 and MK946446). Phylogenetic analysis was assessed through the correlation between sequenced samples, retrieved from vegetation and rabbit, and corresponding tick species *Hyalomma* and *Rhipicephalus* obtained from the GenBank database (Fig 4 and 5).

Fig 4. Phylogenetic tree of the *16S rDNA* sequences of *Hyalomma lusitanicum* isolated from ticks collected from vegetation (isolate 12) and *Rhipicephalus pusillus* isolated from ticks collected from wild rabbits (*Oryctolagus cuniculus*) (isolates 34, 38, 39, 40). The analysis was obtained based on the Neighbor-Joining method with a Tamura-3-parameter model with a discrete Gamma distribution. The characterized species in this study are represented in bold. Sequence names include the GenBank accession number, organism name, host (if mentioned), country of origin and year of collection or submission. The reliability of internal branches was assessed using the bootstrapping method with 1000 replicates.

Fig 5. Phylogenetic tree of the *cytochrome oxidase subunit I* (*COI*) sequences of *Hyalomma lusitanicum* isolated from questing ticks (isolates 8, 12 and 15) and from wild rabbits (*Oryctolagus cuniculus*) (isolates 29 and 42). The analysis was obtained based on the Neighbor-Joining method with Tamura 3 parameters with invariable sites. The characterized species in this study are represented in bold. Sequence names include the GenBank accession number, organism name, host (if mentioned), country of origin and year of collection or submission. The reliability of internal branches was assessed using the bootstrapping method with 1000 replicates.

Ticks and tick-borne pathogens in addax and questing ticks

A total of 116 adult ticks were collected from captured addax. Ticks were morphologically identified as *H. lusitanicum* (76 males and 39 females) and *H. marginatum* (n = 1 male). One individual had *H. lusitanicum* and *H. marginatum*.

Tick-borne pathogens were detected in 20.8% (5/24) of the analysed pooled questing ticks and 27.3% (3/11) of the pooled addax ticks. The pathogen DNA detected in questing ticks corresponded to *Rickettsia* (4.1%) and *Coxiella* (16.7%) species and, in ticks retrieved from addax, *C. burnetii* (27.3%). Sequencing of the *16S rDNA* PCR-positive sample (Isolate 23) to *Rickettsia* spp. resulted in a sequence sharing of 100% nucleotide identity with the *16S rDNA* gene of *R. aeschlimannii* retrieved from *H. rufipes* ticks (GenBank accession number MW394393) and from *H. marginatum* ticks (GenBank accession number MW394392) from Spain (Fig 6). This corresponds to the first record of *R. aeschlimannii* in *H. lusitanicum* free-roaming ticks in vegetation. In addition, BLAST analysis of four tick pools from vegetation (Isolates 13, 14, 16 and 22) and three from addax (Isolates 2A, 6A and 10A) revealed a 99.12 – 100% nucleotide identity to the *IS1111* gene of *C. burnetii* (GenBank accession numbers MG764422, MZ673295 and CP014565). The genetic correlation between isolate samples and *C. burnetii* sequences

retrieved from the GenBank database was assessed through phylogenetic analysis (Fig 7). Concerning pathogen detection, questing ticks and addax tick samples were PCR-negative for *Anaplasma* spp., *Babesia* spp., *Theileria* spp. and CCHFV infectious agents.

Fig 6. Phylogenetic tree of the *16S rDNA* sequence of *Rickettsia aeschlimannii* isolated from *Hyalomma lusitanicum* questing ticks (isolate 23). The analysis was obtained based on the Neighbor-Joining method with a Jukes-Cantor model. The characterized species in this study are represented in bold. Sequence names include the GenBank accession number, organism name, host (if mentioned), country of origin and year of collection or submission. The reliability of internal branches was assessed using the bootstrapping method with 1000 replicates.

Fig 7. Phylogenetic tree of the *IS1111* sequence gene of *Coxiella burnetii* isolated from *Hyalomma lusitanicum* questing ticks (isolate 13, 14, 16, 22) and attached to the Saharan antelope addax (*Addax nasomaculatus*; isolate A2, A6, and A10). The analysis was obtained based on the Neighbor-Joining method with a Kamura 2 parameters model. The characterized species in this study are represented in bold. Sequence names include the GenBank accession number, organism name, host (if mentioned), country of origin and year of collection or submission. The reliability of internal branches was assessed using the bootstrapping method with 1000 replicates.

Discussion

Our results describe the annual distribution of multiple tick species – *H. lusitanicum, R. pusillus*, and *H. aegyptium* – found in vegetation and wild hosts, resulting in significant spatial and seasonal differences. While questing ticks reached their highest abundance in summer, ticks from wild rabbits were most abundant in spring. Ticks from rabbits were density-dependent and showed a positive relationship with questing ticks. Tick network

interactions led to the presence of infectious agents present in both questing and addax ticks, with *C. burnetii* found in both, and *R. aeschlimannii* in questing ticks (Fig 8).
The number of ticks collected in vegetation and hosts varies considerably between seasons and years due to abiotic factors [6,7]. Several tick genera decrease their abundance in winter months and increase it in summer [14]. Our study supports this variation in questing ticks, with the predominant *H. lusitanicum* showing a difference of 1400 individuals when comparing summer with autumn. Accordingly, [38] have documented higher activity from May to July in *H. lusitanicum* adult ticks, which might increase infection rates of hosts in summer in North Africa. In our study, *R. pusillus* was the predominant species followed by *H. lusitanicum* in wild rabbits and the infestation was higher in spring (21 ticks/rabbit) than average (15 ticks/rabbit), as occurred in other Mediterranean areas [16]. *R. pusillus* life cycle is highly dependent on the presence of lagomorphs, increasing rabbit parasitation rates with rabbit densities-dependent effects [2,39]. *H. lusitanicum* network would benefit from rabbit density effects, and its presence in addax might close a cycle of interactions in this species between vegetation, wild rabbits and ungulates as described by [5]. In Mediterranean areas, *H. lusitanicum* in rabbits has been documented to increase in zones where ungulates contact with wild rabbits [16]. Indeed, in our study, this occurs in summer when rabbit densities decrease. Nevertheless, in areas where there is no ungulate-rabbit contact, tick parasitisation increases in spring and autumn, which may be related to the high-density-dependent effect of rabbits. Abiotic factors, such as water bodies, woodlands or scrub cover, as well as biotic ones, such as a high diversity of host species, all contribute to the *H. lusitanicum* cycle [4,40]. On the other hand, *H. aegyptium*, as a questing tick, may be influenced by other potential wild hosts [9,11]. For instance, the density and distribution of tortoises and hedgehogs may help explain the spatial differences observed in *H. aegyptium*, as

scrub cover alone does not seem to account for them. In addition to seasonal variations in tick abundance, annual differences in abiotic conditions, such as dry and humid years, can also affect tick abundance [41,42]. Previous studies have identified rainfall as a key factor for tick abundance, with increasing populations following periods of high cumulative rainfall [43,44]. In fact, significant differences in precipitation between the two studied years resulted in higher questing and on-rabbit ticks in the wetter year (19 and 30% higher, respectively). Current climate change models for the Mediterranean basin predict longer drought periods and more frequent, intense and unpredictable rainfall events[45–47]. Shifts in climate, trending towards warmer temperatures, may alter tick dynamics and seasonal activity patterns, resulting in increased activity in winter [41,48] and potentially the expansion of certain species, such as *H. marginatum* [41]. These climatic changes are likely to make larger regions of southern Europe more favourable for the expansion of North African ticks [49].

Acknowledging some limitations, such as tick-host dynamics and climatic conditions in a given year, we provide evidence of tick interactions between wild hosts and the importance of integrated One Health observational and modelling studies to detect changes in ticks and disease transmission [42,20]. To our knowledge, we report the first worldwide detection of *R. aeschlimannii* in *H. lusitanicum* questing ticks, along with the identification of *C. burnetii*. ~~*R.*~~ *aeschlimannii* belongs to the spotted fever group rickettsiosis, which includes potential zoonotic agents and is associated with the Mediterranean spotted fever-like disease in humans [50,51]. ~~*R.*~~ *aeschlimannii* is most reported in *H. marginatum* ticks, even though it has also been detected in other tick species [12,52,53]. In 2014 – 2017, a 50% prevalence of spotted fever group *Rickettsia* spp. was reported in *H. marginatum* ticks in addax in the same study area [14], with a higher prevalence (27.3%). Prevalence differences may be attributed to intraspecific tick

characteristics, such as internal barriers (e.g. the tick immune system) or co-infection with undetected pathogens, which could influence ticks and their ability to sustain the infection [1].

C. burnetii, the causative agent of Q fever in humans, ranging from mild symptoms, such as fever, to endocarditis in chronic disease stage [54], was detected in 22.6% of questing ticks and 28.6% of addax ticks. *C. burnetii* in wildlife and domestic animals has been described in multiple tick species such as *Ixodes* spp., *Rhipicephalus* spp., *Amblyomma* spp., *Dermacentor* spp. or *Hyalomma* spp. [55]. In Africa, Q fever has a seroprevalence of 8% in humans; higher in agricultural countries (e.g. Algeria or Egypt with 15% and 16% seroprevalence, respectively) [56,55]. In cattle and small ruminants from Africa, antibody seroprevalence against *C. burnetii* ranges from 18% to 55% and 11% to 33%, respectively [56]. In addition to its importance in human health, *C. burnetii* infection in sheep can lead to abortion and cause reproductive losses in wild ungulates, such as red deer [57], suggesting that addax might also be affected. The absence of *Anaplasma* spp., *Babesia* spp., *Theileria* spp. and CCHFV in *H. lusitanicum* stands in contrast to the prevalence of these pathogens in *H. marginatum* found within the addax population of the studied area [14]. These differences may be attributed to factors such as a smaller sample size, intraspecific tick characteristics, or low-level pathogen infection in the analysed ticks, which may hinder pathogen detection and lead to an underestimation of pathogen prevalence [58].

The presence of *R. aeschlimannii* and *C. burnetii* in *H. lusitanicum* questing ticks suggests that these pathogens may also be present in wild rabbits and ungulates. This possibility is further supported by the detection of *C. burnetii* in other Mediterranean populations [16]. The wide diversity of potential hosts and their close contact with livestock and humans in Maamora forest, which may become infested by questing ticks, increases the

opportunities for pathogen transmission and the possible infection of wild and domestic animals [59,52,60]. This also poses a risk to humans [50], thereby favouring the emerging or re-emerging of zoonotic infections in North Africa [20].

This study presents some limitations. The timeframe of the study, a little more than a year, may limit the ability to depict and fully comprehend the tick-host dynamics. In addition, due to budget constraints, we were unable to analyse tick-borne pathogens in ticks infesting rabbits, which could provide valuable insights into disease transmission dynamics. Nevertheless, this study paves the way for new research, highlighting the intricate network interactions of ticks from off-host to wild hosts and their pathogens.

To understand how future climate scenarios may impact the distribution and ecology of North African ticks and tick-borne diseases in North Africa, long-term studies are needed. Prolonged fieldwork is required to elucidate the complex nature of ticks, including questing-wild hosts-pathogen relationships. Enhanced surveillance is necessary to detect and control North African ticks originating from the importation of animals (livestock) and exotic pet trade (e.g. tortoises) to prevent the inadvertent establishment of exotic North African tick species in Europe. In this line, the trans-Mediterranean movement of ticks between Europe and North Africa presents a serious challenge, not only to human health, but also to animals and ecosystems. Conservation and management programs could bring together researchers and policymakers from both European and North African countries, fostering collaboration through a One Health approach.

Fig 8. Summary of this study major findings. Representation of encountered ticks (*Hyalomma lusitanicum*, *Hyalomma marginatum*, and *Rhipicephalus pusillus*) and tick-borne pathogens (*Rickettsia aeschlimannii*, and *Coxiella burnetii*) in questing ticks and investigated hosts (wild rabbit and addax). In addition, the highest number of ticks

collected from vegetation and rabbits according to sampling zone and period are also represented.

Ethics statement

Acknowledgements

We thank Hassan Belhajjamia, Abdessalam Belhajjamia and Bouhali Kaddouri for their field assistance in the collection of ticks. We would also like to thank Greg Trollip and Jacob Mwanzia for their support and interest in wild species conservation.

Ethics approval

Not required

Author contributions

MR: data acquisition and analysis, methodology design, research, writing and editing of the manuscript; AS: data acquisition and analysis, writing and editing of the manuscript; RV: methodology design, reviewed and edited the final manuscript; DV: data acquisition; OR: data acquisition, reviewed and edited the final manuscript; GF: data acquisition, reviewed and edited the final manuscript; JI: data acquisition, reviewed and edited the final manuscript; CG: study conceptualization, reviewed and edited the final manuscript; JF: study conceptualization, supervised research, edited and approved the final manuscript. All authors have read and agreed to the published version of the manuscript

Funding

This research was partly supported by the 2022-GRIN-34227 grant, funded by the University of Castile-La Mancha (UCLM), Spain and EU-FEDER. R. Vaz-Rodrigues was supported by a doctoral contract (2022-PRED-20675), from UCLM and co-financed by the European Social Fund (ESF).

Conflict of interest statement

The authors declare that the research was conducted in the absence of any commercial or financial relationships that could be construed as a potential conflict of interest.

References

1. de la Fuente J, Contreras M, Estrada-Peña A, Cabezas-Cruz A. Targeting a global health problem: Vaccine design and challenges for the control of tick-borne diseases. Vaccine. 2017;35: 5089–5094. doi:10.1016/j.vaccine.2017.07.097

2. Estrada-Peña A, Mihalca AD, Petney TN. Ticks of Europe and North Africa: A Guide to Species Identification. Springer; 2017.

3. Sonenshine DE, Roe RM. Biology of Ticks Volume 1. OUP USA; 2013.

4. Allan BF, Dutra HP, Goessling LS, Barnett K, Chase JM, Marquis RJ, et al. Invasive honeysuckle eradication reduces tick-borne disease risk by altering host dynamics. Proc Natl Acad Sci U S A. 2010;107: 18523–18527. doi:10.1073/pnas.1008362107

5. Valcárcel F, Elhachimi L, Vilá M, Tomassone L, Sánchez M, Selles SMA, et al. Emerging *Hyalomma lusitanicum*: From identification to vectorial role and integrated control. Med Vet Entomol. 2023;37: 425–459. doi:10.1111/mve.12660

6. Daniel M, Cerný V, Dusbábek F, Honzáková E, Olejnícek J. Influence of microclimate on the life cycle of the common tick *Ixodes ricinus* (L.) in thermophilic oak forest. Folia Parasitol (Praha). 1976;23: 327–342.

7. Medlock JM, Hansford KM, Bormane A, Derdakova M, Estrada-Peña A, George J-C, et al. Driving forces for changes in geographical distribution of *Ixodes ricinus* ticks in Europe. Parasit Vectors. 2013;6: 1. doi:10.1186/1756-3305-6-1

8. Yessinou RE, Koumassou A, Galadima HB, Nanoukon-Ahigan H, Farougou S, Pfeffer M. Tick Diversity and Distribution of Pathogen in Ticks Collected from Wild Animals and Vegetation in Africa. Pathogens. 2025;14: 116. doi:10.3390/pathogens14020116

9. Segura A, Rodríguez O, Ruiz-Fons F, Acevedo P. Tick parasitism in the Mediterranean spur-thighed tortoise in the Maamora forest, Morocco. Ticks Tick-Borne Dis. 2019;10: 286–289. doi:10.1016/j.ttbdis.2018.11.002

10. Norte AC, Harris DJ, Silveira D, Nunes CS, Núncio MS, Martínez EG, et al. Diversity of microorganisms in *Hyalomma aegyptium* collected from spur-thighed tortoise (*Testudo graeca*) in North Africa and Anatolia. Transbound Emerg Dis. 2022;69: 1951–1962. doi:10.1111/tbed.14188

11. Segura A, Rafael M, Vaz-Rodrigues R, Rodríguez O, Gortázar C, de la Fuente J. Tick infestation in spur-thighed tortoise population: a pilot study for unraveling epidemiological patterns and demographic consequences. Exp Appl Acarol. 2023 [cited 21 Nov 2023]. doi:10.1007/s10493-023-00863-7

12. Keysary A, Eremeeva ME, Leitner M, Din AB, Wikswo ME, Mumcuoglu KY, et al. Spotted Fever Group Rickettsiae in Ticks Collected from Wild Animals in Israel. Am J Trop Med Hyg. 2011;85: 919–923. doi:10.4269/ajtmh.2011.10-0623

13. Said Y, Lahmar S, Dhibi M, Rjeibi MR, Jdidi M, Gharbi M. First survey of ticks, tick-borne pathogens (*Theileria*, *Babesia*, *Anaplasma* and *Ehrlichia*) and *Trypanosoma evansi* in protected areas for threatened wild ruminants in Tunisia. Parasitol Int. 2021;81: 102275. doi:10.1016/j.parint.2020.102275

14. Rodríguez O, de la Fuente G, Fernández de Mera IG, Vaz-Rodrigues R, Gortázar C, de la Fuente J. The Saharan antelope addax (*Addax nasomaculatus*) as a host for *Hyalomma marginatum*, tick vector of Crimean-Congo hemorrhagic fever virus. Ticks Tick-Borne Dis. 2022;13: 102034. doi:10.1016/j.ttbdis.2022.102034

15. Loftis AD, Reeves WK, Szumlas DE, Abbassy MM, Helmy IM, Moriarity JR, et al. Population Survey of Egyptian Arthropods for Rickettsial Agents. Ann N Y Acad Sci. 2006;1078: 364–367. doi:10.1196/annals.1374.072

16. Sánchez M, Valcárcel F, González J, González MG, Martín-Hernández R, Tercero JM, et al. Seasonality of *Coxiella burnetii* among Wild Rabbits (*Oryctolagus cuniculus*) and the *Hyalomma lusitanicum* (Acari: Ixodidae) in a Meso-Mediterranean Ecosystem. Pathogens. 2022;11: 36. doi:10.3390/pathogens11010036

17. Ruiz-Fons F, Fernández-de-Mera IG, Acevedo P, Gortázar C, de la Fuente J. Factors Driving the Abundance of *Ixodes ricinus* Ticks and the Prevalence of Zoonotic *I. ricinus*-Borne Pathogens in Natural Foci. Appl Environ Microbiol. 2012;78: 2669–2676. doi:10.1128/AEM.06564-11

18. Wikel SK. Ticks and Tick-Borne Infections: Complex Ecology, Agents, and Host Interactions. Vet Sci. 2018;5: 60. doi:10.3390/vetsci5020060

19. d’Oliveira C, van der Weide M, Jacquiet P, Jongejan F. Detection of *Theileria annulata* by the PCR in ticks (Acari: Ixodidae) collected from cattle in Mauritania. Exp Appl Acarol. 1997;21: 279–291. doi:10.1023/A:1018455223462

20. Abdelbaset AE, Kwak ML, Nonaka N, Nakao R. Human-biting ticks and zoonotic tick-borne pathogens in North Africa: diversity, distribution, and trans-Mediterranean public health challenges. One Health Amst Neth. 2023;16: 100547. doi:10.1016/j.onehlt.2023.100547

21. Vatansever Z, Gargili A, Aysul NS, Sengoz G, Estrada-Peña A. Ticks biting humans in the urban area of Istanbul. Parasitol Res. 2008;102: 551–553. doi:10.1007/s00436-007-0809-z

22. Bursali A, Tekin S, Keskin A, Ekici M, Dundar E. Species diversity of ixodid ticks feeding on humans in Amasya, Turkey: seasonal abundance and presence of Crimean-Congo hemorrhagic fever virus. J Med Entomol. 2011;48: 85–93. doi:10.1603/me10034

23. Fennane M, Rejdali M. The world largest cork oak Maamora forest: challenges and the way ahead. Flora Mediterr. 2015;25: 277–285. doi:10.7320/FlMedit25SI.277

24. IUCN. IUCN SSC Antelope Specialist Group. *Addax nasomaculatus*. The IUCN Red List of Threatened Species 2016: e.T512A50180603. IUCN. 2016 [cited 29 Nov 2023]. doi:10.2305/IUCN.UK.2016-2.RLTS.T512A50180603.en

25. Segura A, Rotger A, Rodríguez-Caro RC. Hidden Threats to Persistence: Changes in Population Structure Can Affect Well-Preserved Spur-Thighed Tortoise Populations. Herpetologica. 2025;81: 23–33. doi:10.1655/Herpetologica-D-23-00066

26. Filahi S, Tanarhte M, Mouhir L, El Morhit M, Tramblay Y. Trends in indices of daily temperature and precipitations extremes in Morocco. Theor Appl Climatol. 2016;124: 959–972. doi:10.1007/s00704-015-1472-4

27. Said L, Assmaa A, Najib G, Gmira N. Contribution à l'évaluation de la pression pastorale dans la forêt de la Maamora. Parcours forestiers et surpâturage. Nat Tecnol. 2014; 39–50.

28. Coimbra-Dores MJ, Maia-Silva M, Marques W, Oliveira AC, Rosa F, Dias D. Phylogenetic insights on Mediterranean and Afrotropical *Rhipicephalus* species (Acari: Ixodida) based on mitochondrial DNA. Exp Appl Acarol. 2018;75: 107–128. doi:10.1007/s10493-018-0254-y

29. Moraga Fernández A, Ortiz JA, Jabbar A, Ghafar A, Cabezas-Cruz A, de la Fuente G, et al. Fatal cases of bovine anaplasmosis in a herd infected with different *Anaplasma marginale* genotypes in southern Spain. Ticks Tick Borne Dis. 2022; 101864–101864.

30. Rjeibi MR, Amairia S, Mhadhbi M, Rekik M, Gharbi M. Detection and molecular identification of *Anaplasma phagocytophilum* and *Babesia* spp. infections in *Hyalomma aegyptium* ticks in Tunisia. Arch Microbiol. 2022;204: 385. doi:10.1007/s00203-022-02995-7

31. Moraga-Fernández A, Ruiz-Fons F, Habela MA, Royo-Hernández L, Calero-Bernal R, Gortazar C, et al. Detection of new Crimean-Congo haemorrhagic fever virus genotypes in ticks feeding on deer and wild boar, Spain. Transbound Emerg Dis. 2021;68: 993–1000. doi:10.1111/tbed.13756

32. Moraga-Fernández A, Chaligiannis I, Cabezas-Cruz A, Papa A, Sotiraki S, de la Fuente J, et al. Molecular identification of spotted fever group Rickettsia in ticks collected from dogs and small ruminants in Greece. Exp Appl Acarol. 2019;78: 421–430. doi:10.1007/s10493-019-00392-2

33. Fernández de Mera IG, Zivkovic Z, Bolaños M, Carranza C, Pérez-Arellano JL, Gutiérrez C, et al. *Rickettsia massiliae* in the Canary Islands. Emerg Infect Dis. 2009;15: 1869–1870. doi:10.3201/eid1511.090681

34. Roux V, Rydkγna E, Eremeeva M, Raoult D. Citrate Synthase Gene Comparison, a New Tool for Phylogenetic Analysis, and Its Application for the Rickettsiae†. Int J Syst Evol Microbiol. 1997;47: 252–261. doi:10.1099/00207713-47-2-252

35. Bonsergent C, Vittecoq M, Leray C, Burkart L, McCoy KD, Malandrin L. Characterization and diversity of *Babesia* sp. YLG, a new member of the Peircei

group infecting Mediterranean yellow-legged gulls (*Larus michahellis*). Ticks Tick-Borne Dis. 2022;13: 101852. doi:10.1016/j.ttbdis.2021.101852

36. Akaike H. A new look at the statistical model identification. IEEE Trans Autom Control. 1974;19: 716–723. doi:10.1109/TAC.1974.1100705

37. R Core Team. R: The R Project for Statistical Computing. Vienna, Austria: R Foundation for Statistical Computing; 2023. Available: https://www.R-project.org/

38. Estrada-Peña A, Bouattour A, J-L C, Walker A. Ticks of Domestic Animals in Africa. A Guide to Identification of Species. 2004.

39. Castro-Scholten S, Caballero-Gómez J, Martínez R, Nadales-Martín BJ, Cano-Terriza D, Jiménez-Martín D, et al. Occurrence of *Coxiella burnetii* in wild lagomorphs and their ticks in Spanish Mediterranean ecosystems. Zoonoses Public Health. 2024;71: 549–559. doi:10.1111/zph.13155

40. Peralbo-Moreno A, Baz-Flores S, Cuadrado-Matías R, Barroso P, Triguero-Ocaña R, Jiménez-Ruiz S, et al. Environmental factors driving fine-scale ixodid tick abundance patterns. Sci Total Environ. 2022;853: 158633. doi:10.1016/j.scitotenv.2022.158633

41. Estrada-Peña A, Venzal JM. Climate niches of tick species in the Mediterranean region: modeling of occurrence data, distributional constraints, and impact of climate change. J Med Entomol. 2007;44: 1130–1138. doi:10.1603/0022-2585(2007)44[1130:cnotsi]2.0.co;2

42. Ogden NH, Ben Beard C, Ginsberg HS, Tsao JI. Possible Effects of Climate Change on Ixodid Ticks and the Pathogens They Transmit: Predictions and Observations. J Med Entomol. 2021;58: 1536–1545. doi:10.1093/jme/tjaa220

43. Keesing F, Ostfeld RS, Young TP, Allan BF. Cattle and rainfall affect tick abundance in central Kenya. Parasitology. 2018;145: 345–354. doi:10.1017/S003118201700155X

44. Bugmyrin SV, Bespyatova LA, Korotkov YuS. Long-term dynamics of *Ixodes persulcatus* (Acari: Ixodidae) abundance in the north–west of its range (Karelia, Russia). Exp Appl Acarol. 2019;77: 229–240. doi:10.1007/s10493-019-00342-y

45. Intergovernmental Panel on Climate Change (IPCC). Climate Change 2013 – The Physical Science Basis: Working Group I Contribution to the Fifth Assessment Report of the Intergovernmental Panel on Climate Change. Cambridge: Cambridge University Press; 2014. doi:10.1017/CBO9781107415324

46. Polade SD, Gershunov A, Cayan DR, Dettinger MD, Pierce DW. Precipitation in a warming world: Assessing projected hydro-climate changes in California and other Mediterranean climate regions. Sci Rep. 2017;7: 10783. doi:10.1038/s41598-017-11285-y

47. Driouech F, Stafi H, Khouakhi A, Moutia S, Badi W, ElRhaz K, et al. Recent observed country-wide climate trends in Morocco. Int J Climatol. 2021;41: E855–E874. doi:10.1002/joc.6734

48. Gray JS, Dautel H, Estrada-Peña A, Kahl O, Lindgren E. Effects of climate change on ticks and tick-borne diseases in Europe. Interdiscip Perspect Infect Dis. 2009;2009: 593232. doi:10.1155/2009/593232

49. Buczek AM, Buczek W, Buczek A, Bartosik K. The Potential Role of Migratory Birds in the Rapid Spread of Ticks and Tick-Borne Pathogens in the Changing Climatic and Environmental Conditions in Europe. Int J Environ Res Public Health. 2020;17: 2117. doi:10.3390/ijerph17062117

50. Raoult D, Fournier P-E, Abboud P, Caron F. First Documented Human *Rickettsia aeschlimannii* Infection. Emerg Infect Dis. 2002;8: 748–749. doi:10.3201/eid0807.010480

51. Germanakis A, Chochlakis D, Angelakis E, Tselentis Y, Psaroulaki A. *Rickettsia aeschlimannii* infection in a Man, Greece. Emerg Infect Dis. 2013;19. doi:10.3201/eid1907.130232

52. Chisu V, Leulmi H, Masala G, Piredda M, Foxi C, Parola P. Detection of *Rickettsia hoogstraalii*, *Rickettsia helvetica*, *Rickettsia massiliae*, *Rickettsia slovaca* and *Rickettsia aeschlimannii* in ticks from Sardinia, Italy. Ticks Tick-Borne Dis. 2017;8: 347–352. doi:10.1016/j.ttbdis.2016.12.007

53. Chochlakis D, Ioannou I, Sandalakis V, Dimitriou T, Kassinis N, Papadopoulos B, et al. Spotted Fever Group Rickettsiae in Ticks in Cyprus. Microb Ecol. 2012;63: 314–323. doi:10.1007/s00248-011-9926-4

54. Maurin M, Raoult D. Q Fever. Clin Microbiol Rev. 1999;12: 518–553. doi:10.1128/cmr.12.4.518

55. Eldin C, Mélenotte C, Mediannikov O, Ghigo E, Million M, Edouard S, et al. From Q Fever to *Coxiella burnetii* Infection: a Paradigm Change. Clin Microbiol Rev. 2016;30: 115–190. doi:10.1128/cmr.00045-16

56. Vanderburg S, Rubach MP, Halliday JEB, Cleaveland S, Reddy EA, Crump JA. Epidemiology of *Coxiella burnetii* Infection in Africa: A OneHealth Systematic Review. PLoS Negl Trop Dis. 2014;8: e2787. doi:10.1371/journal.pntd.0002787

57. González-Barrio D, Almería S, Caro MR, Salinas J, Ortiz JA, Gortázar C, et al. *Coxiella burnetii* Shedding by Farmed Red Deer (Cervus elaphus). Transbound Emerg Dis. 2015;62: 572–574. doi:10.1111/tbed.12179

58. Yang S, Rothman RE. PCR-based diagnostics for infectious diseases: uses, limitations, and future applications in acute-care settings. Lancet Infect Dis. 2004;4: 337–348. doi:10.1016/S1473-3099(04)01044-8

59. Kleinerman G, Baneth G, Mumcuoglu KY, van Straten M, Berlin D, Apanaskevich DA, et al. Molecular Detection of *Rickettsia africae*, *Rickettsia aeschlimannii*, and *Rickettsia sibirica mongolitimonae* in Camels and *Hyalomma* spp. ticks from Israel. Vector-Borne Zoonotic Dis. 2013;13: 851–856. doi:10.1089/vbz.2013.1330

60. Abdelkadir K, Palomar AM, Portillo A, Oteo JA, Ait-Oudhia K, Khelef D. Presence of *Rickettsia aeschlimannii*, '*Candidatus* Rickettsia barbariae' and *Coxiella burnetii* in ticks from livestock in Northwestern Algeria. Ticks Tick-Borne Dis. 2019;10: 924–928. doi:10.1016/j.ttbdis.2019.04.018

Figure1 Click here to access/download;Figure;Fig 1.tif

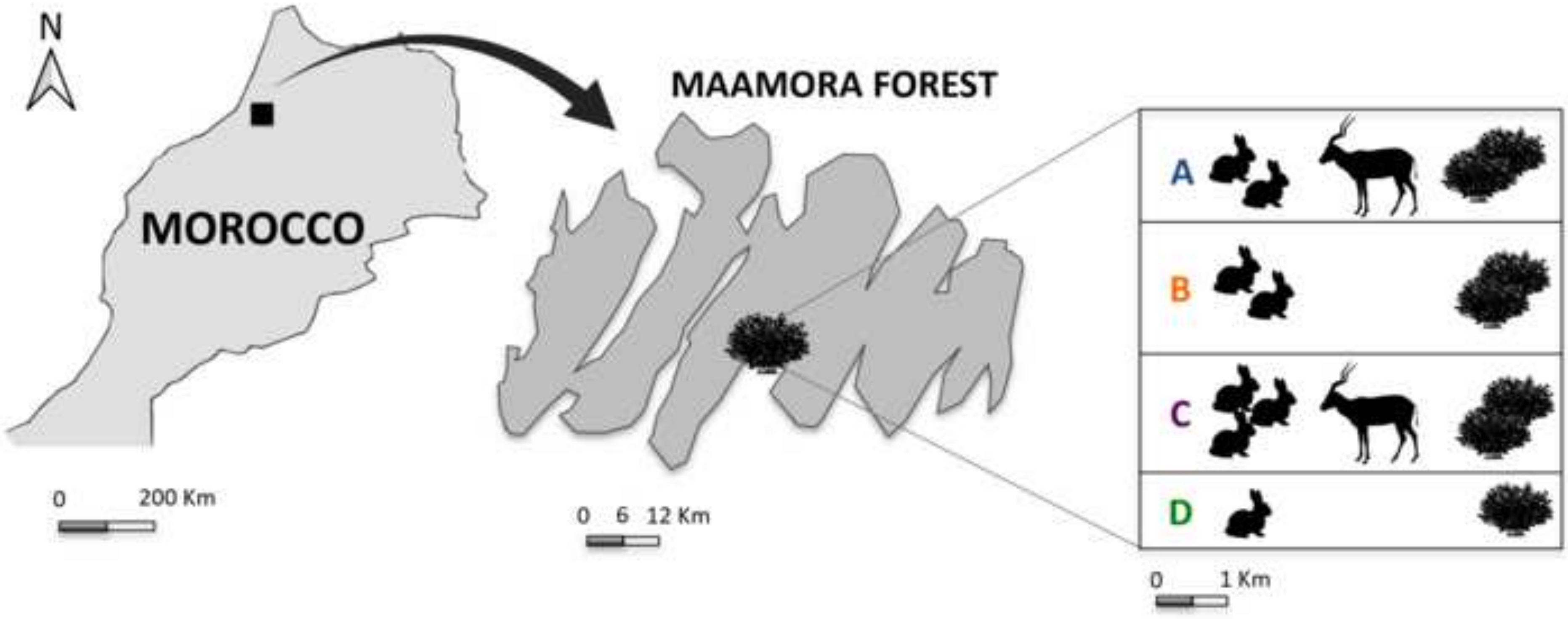

Figure2 Click here to access/download;Figure;Fig 2.tif

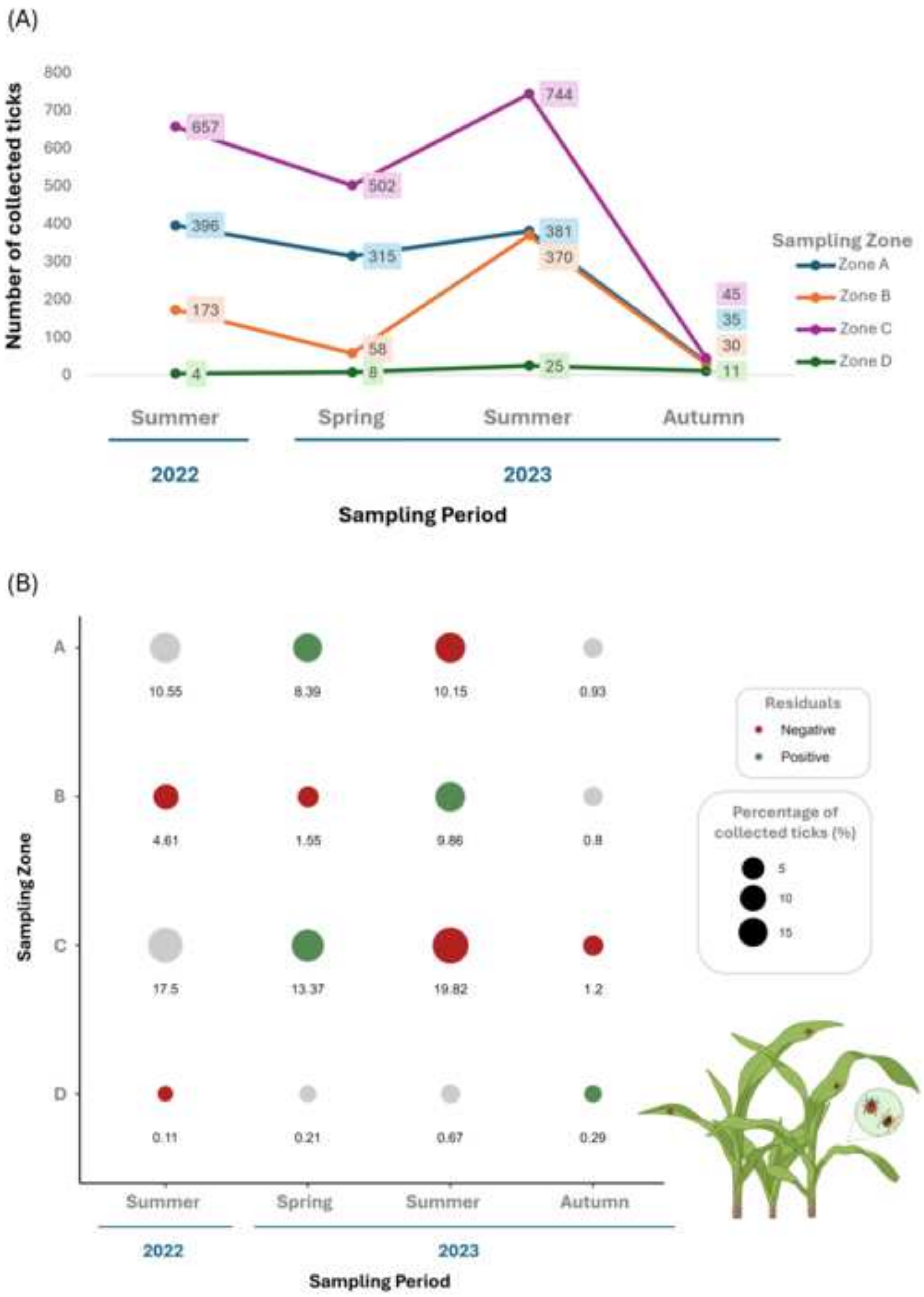

Figure3

Click here to access/download;Figure;Fig 3.tif

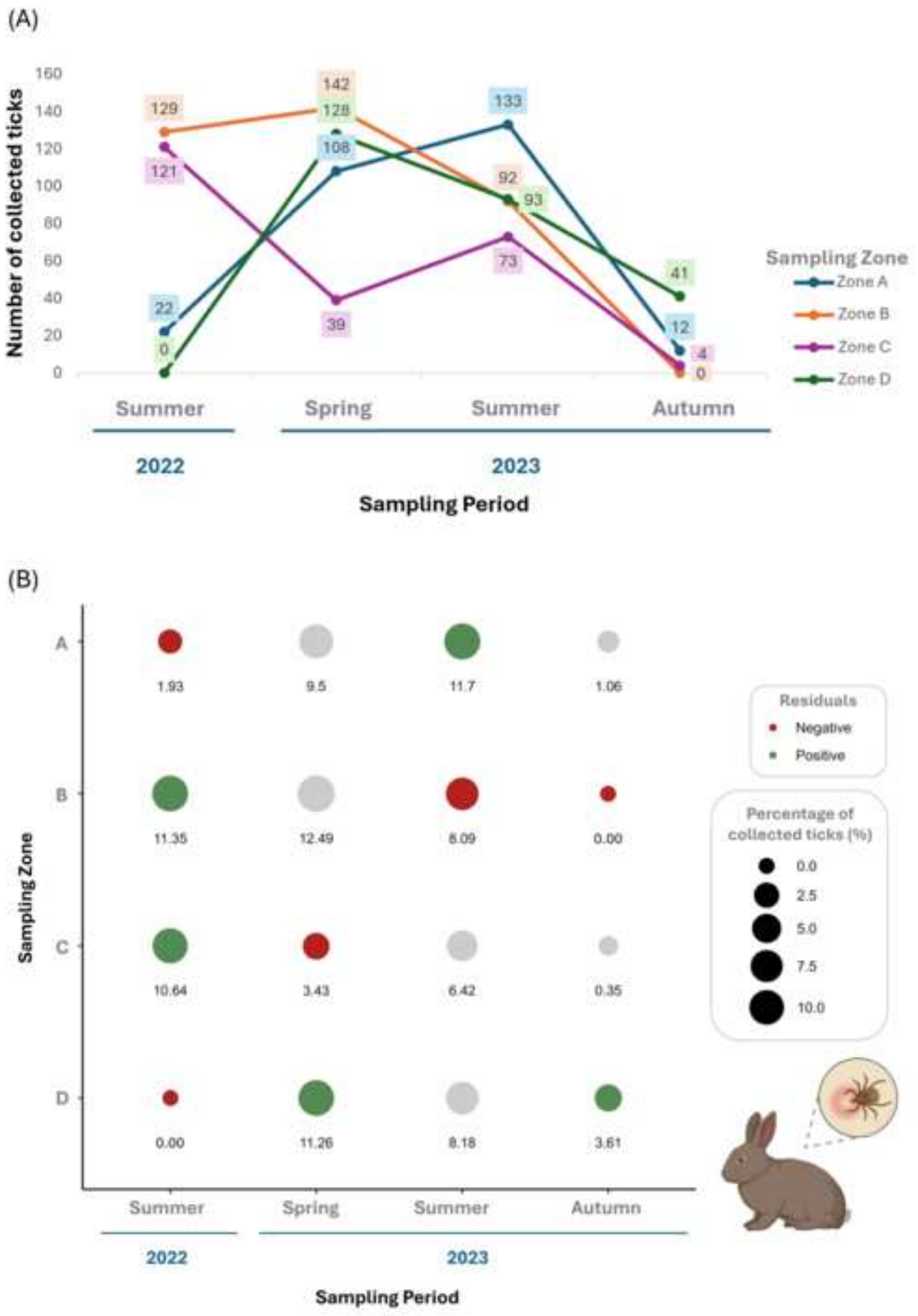

 Click here to access/download;Figure;Fig S2.tif

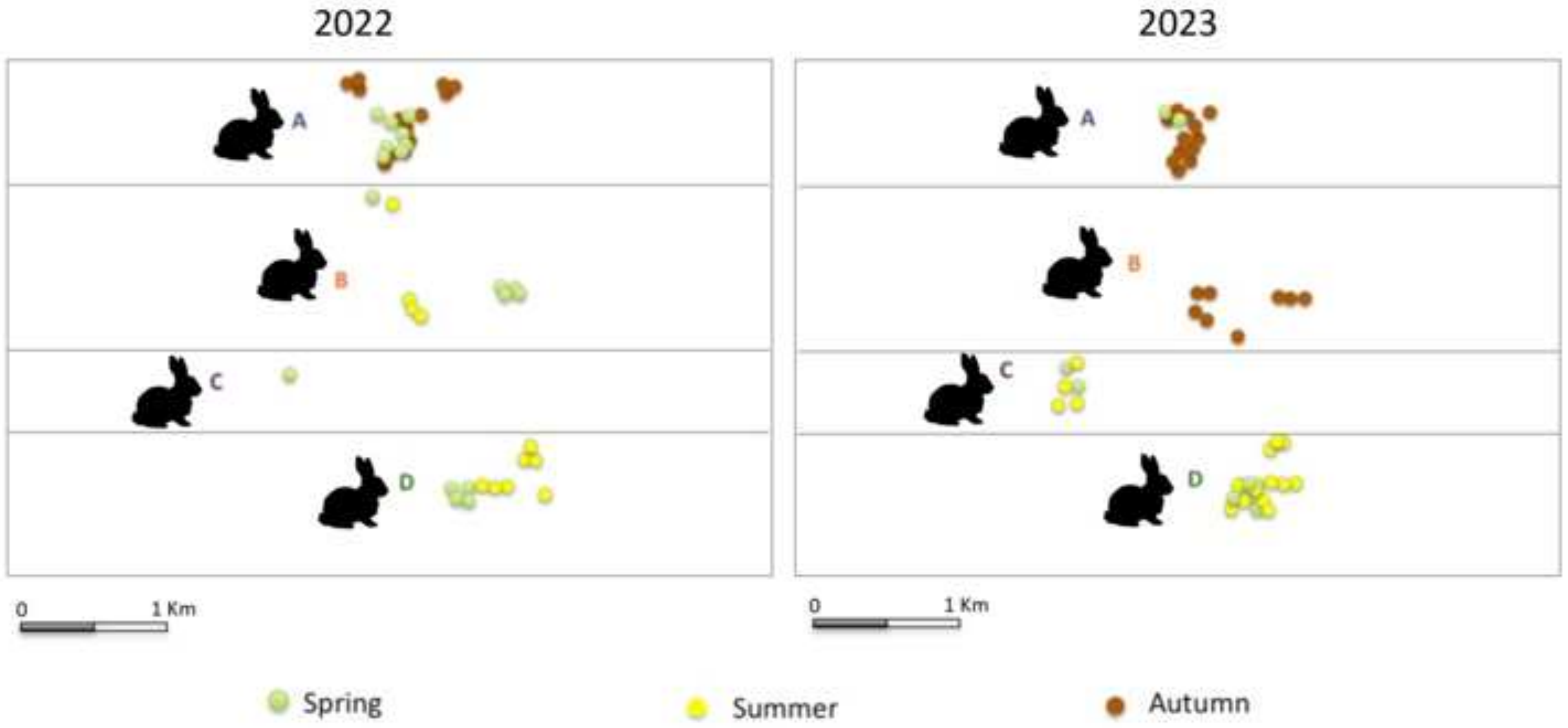

 Click here to access/download;Figure;Fig 4.tif

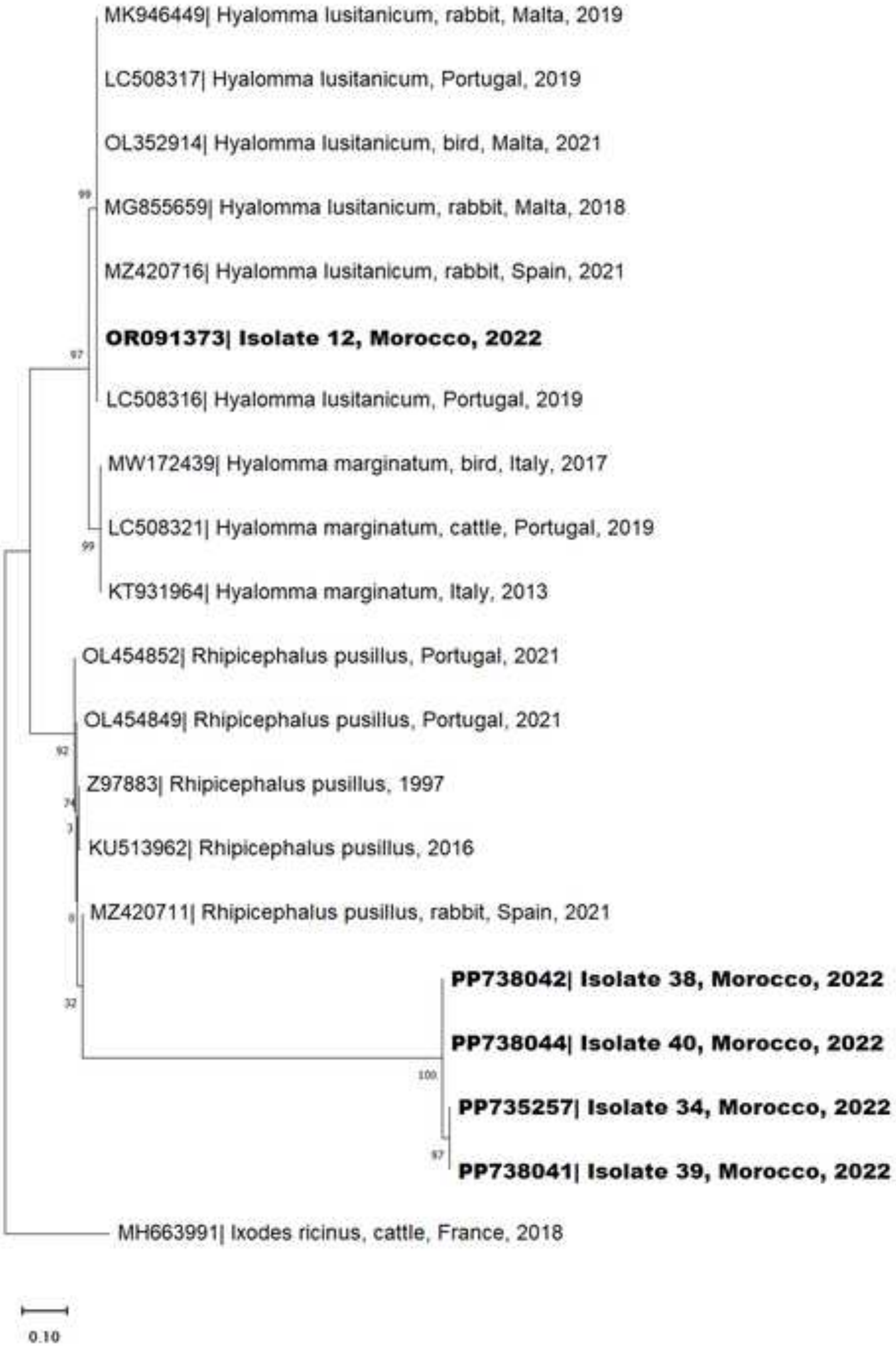

Figure5

Click here to access/download;Figure;Fig 5.tif

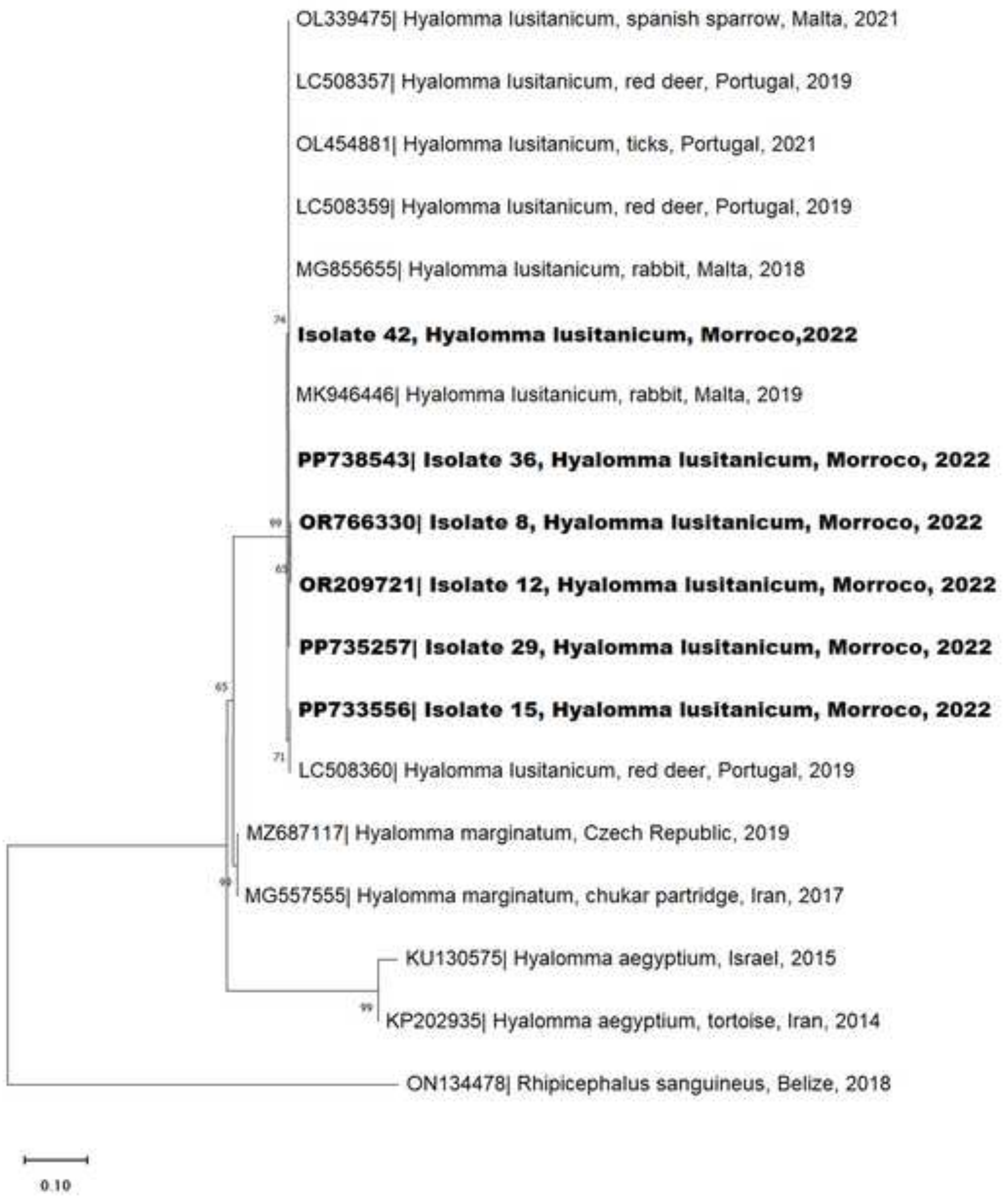

Click here to access/download;Figure;Fig 6.tif

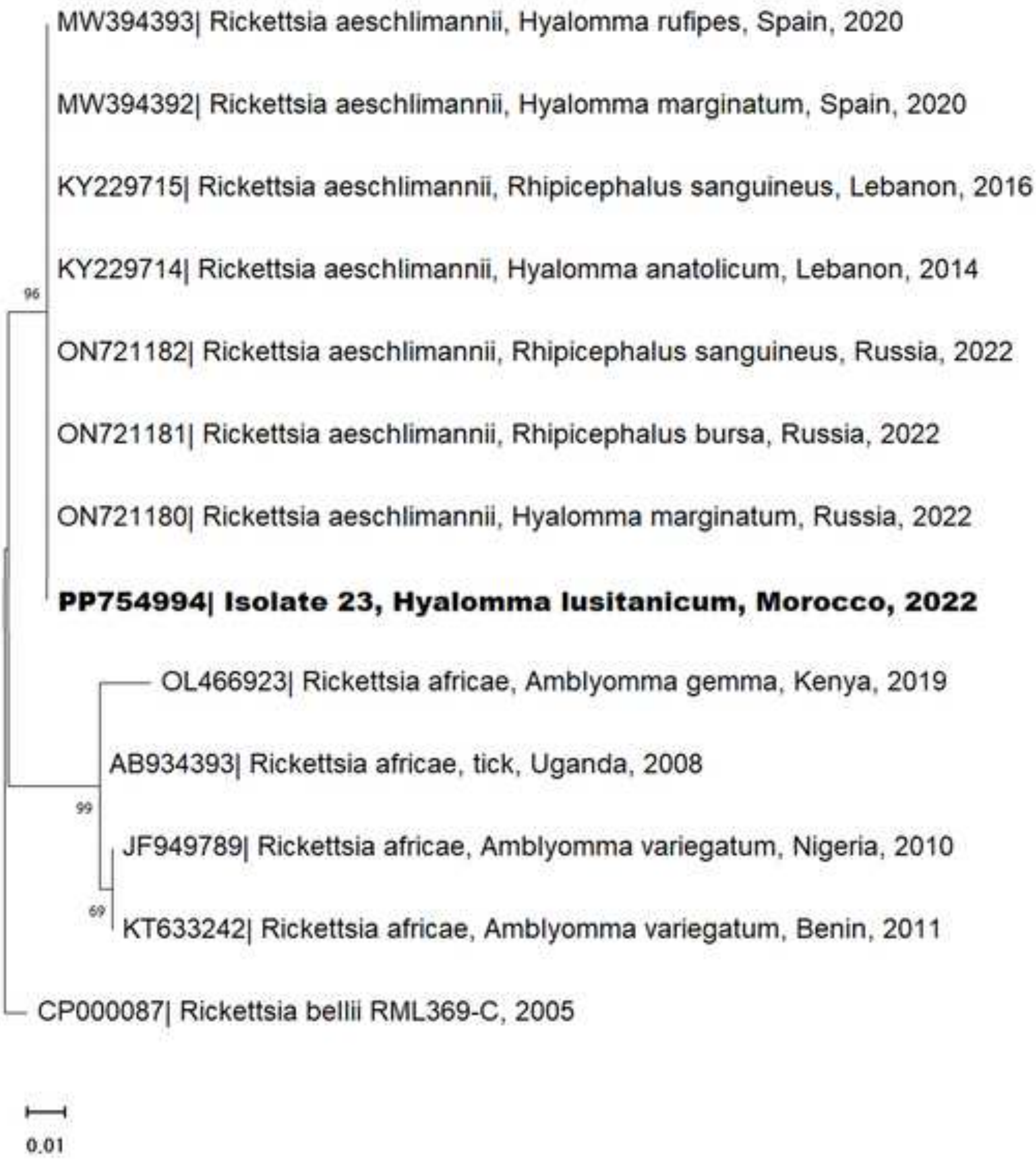

 Click here to access/download;Figure;Fig 7.tif

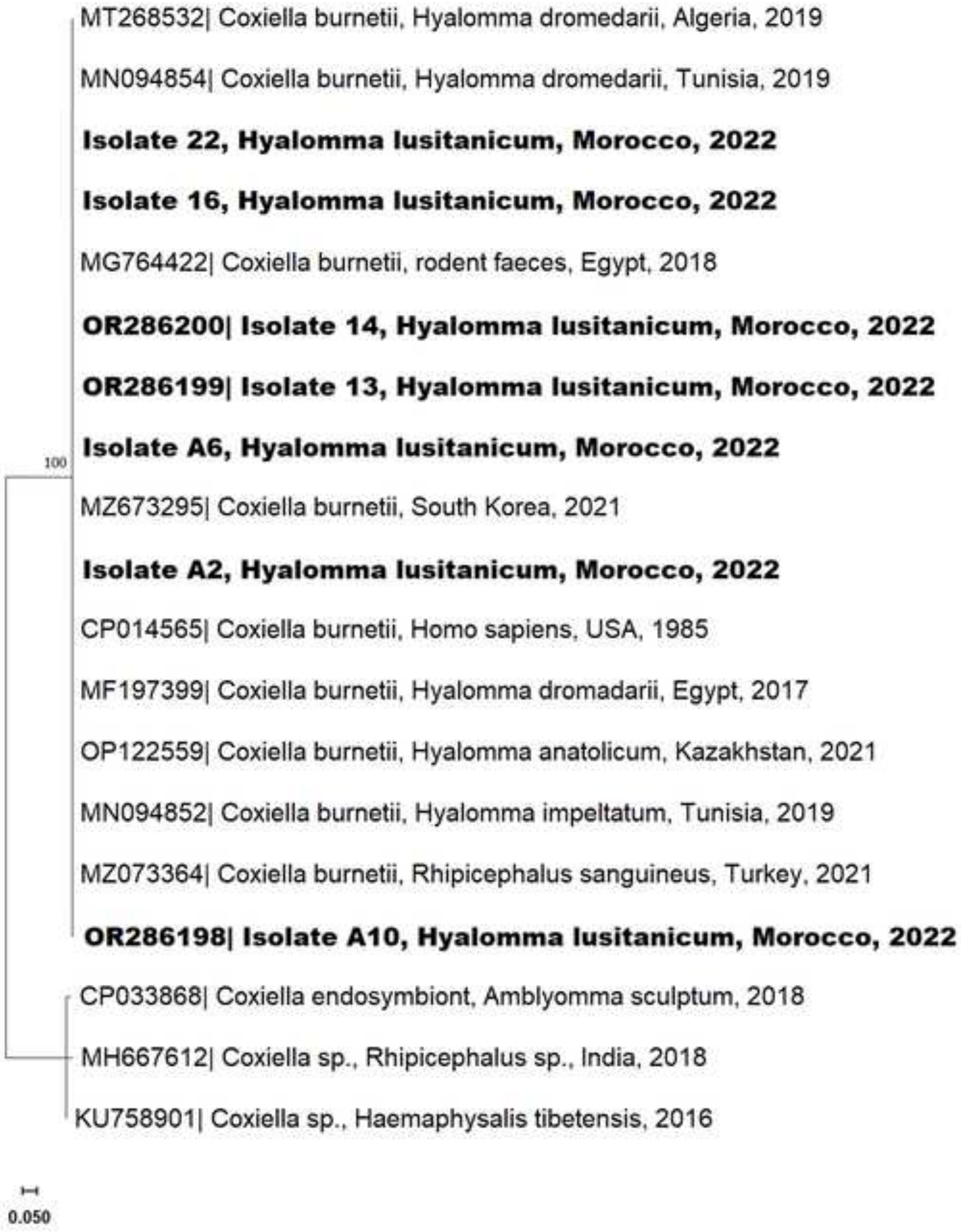

Figure8

Click here to access/download;Figure;Fig 8.tif

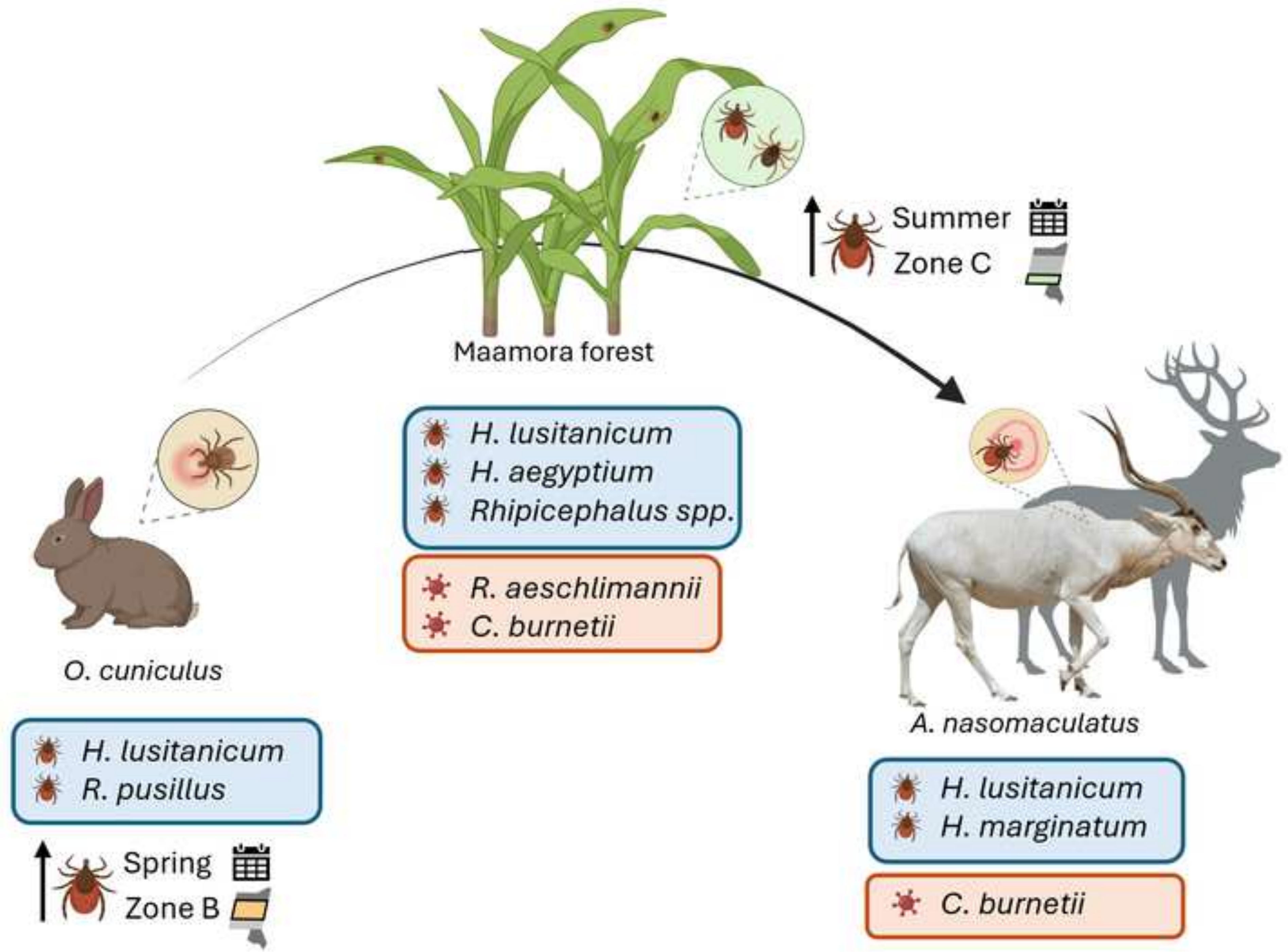

FigureS1

Click here to access/download;Figure;Fig S1.tif

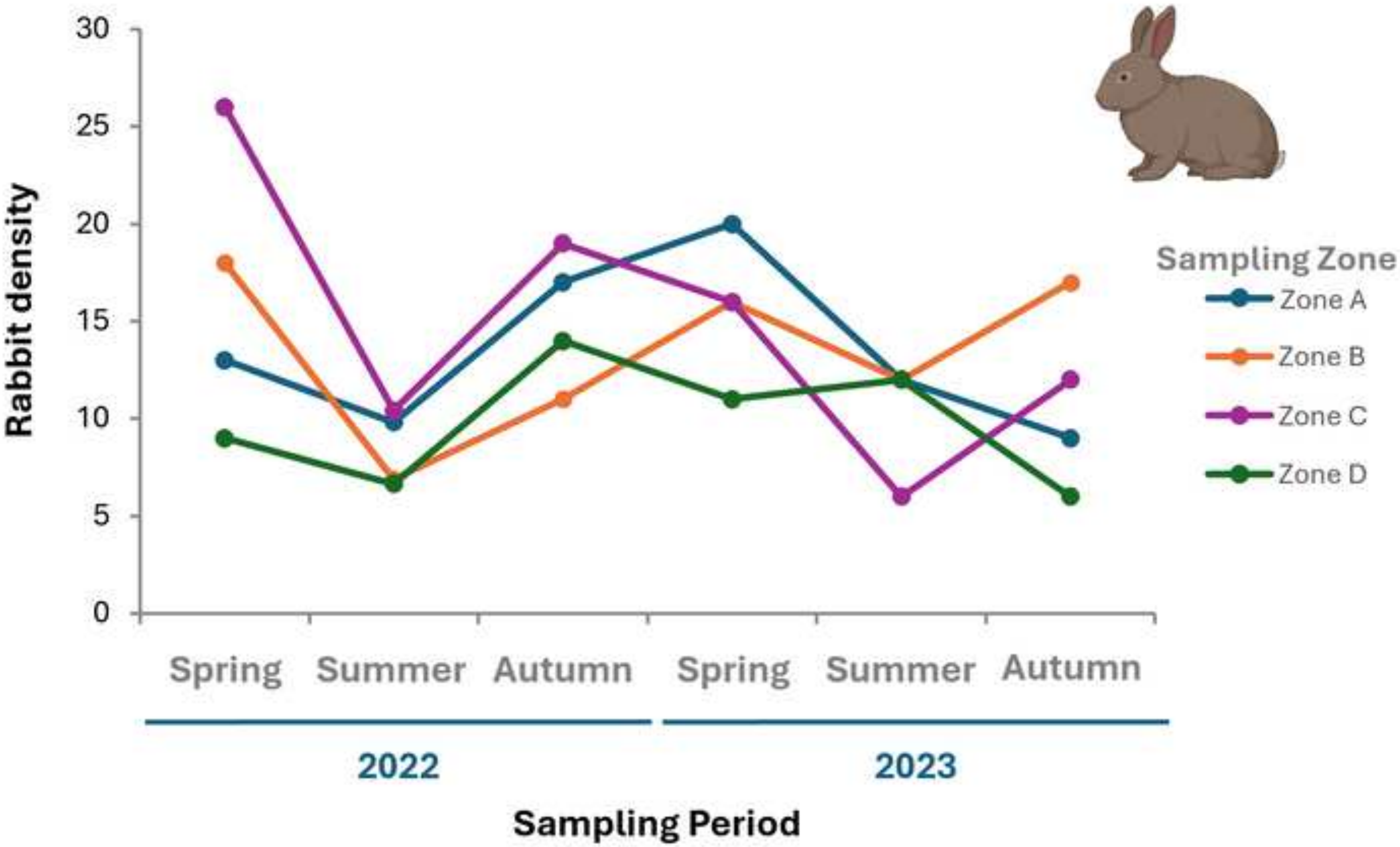

Click here to access/download
Supporting Information - Compressed/ZIP File Archive
Suplementary data.zip
