# Supplementary material for: Tick-wildlife host-pathogen network interactions in Northern Africa
Source: PLoS One. 2025 Jul 15;20(7):e0327313. doi: 10.1371/journal.pone.0327313 (PMC12262905; doi:10.1371/journal.pone.0327313)
Supplement: Attachment — Submitted filename: Response to Reviewers.docx [file pone.0327313.s007.docx]

Dear Editor,

Please, find enclosed a revised version of the manuscript entitled “**Tick-wildlife host-pathogen network interactions in Northern Africa**”.

We appreciate the positive comments from the reviewers, and their great contribution to the revised version of the manuscript. We have made the revision to adapt all the suggestions, both from the reviews and the editor. We believe that the new version improves the previous one and can constitute a good contribution to increase the knowledge on the ecological and epidemiological significance of tick-host-pathogen interactions.

Please feel free to request any additional information.

Yours sincerely,

Marta Rafael

**Reviewer #1:**

* Introduction

- The introduction provides sufficient background and well-written study objectives. However, it would be better to add how to investigate with technical methods in the introduction.

Methods to achieve the proposed objectives have been added to the introduction as: “i) determine the abundance and diversity of questing ticks by flagging and ticks collected from dead rabbits seasonally, ii) determine density-dependent and environmental effects in questing ticks and ticks from wild rabbits, and iii) determine tick-borne pathogens circulating in questing ticks and those infecting addax. Tick-borne pathogens were detected by conventional PCR techniques, aimed at *Anaplasma* spp., *Babesia* spp., *C. burnetii*, *Rickettsia* spp., *Theileria* spp. and CCHFV, previously described pathogens in Africa [2,11,19,20] and in the study area [11,14], followed by a phylogenetic analysis of positive results.” (see lines 108-115 in manuscript with track changes).

- Line 74: Hyalomma (H.) aegyptium

Thank you very much, it has been corrected.

- Line 109: This sentence is quite objective.

The sentence has been rewritten: “This study results provide information to advance in a One-Health approach aimed at preventing the transmission of zoonotic diseases across trans-Mediterranean regions impacting both Europe and Northern Africa [20].”

- Line 96: Why did you choose only wild rabbits?

Thank you very much for this question. We were expecting the presence of *Hyalomma lusitanicum* and *H. marginatum* ticks, which had been previously detected in the study area. Wild rabbits were selected due to their influence in the tick’s life cycle, for being highly abundant and for its long-term monitoring program in the area. In the future, we are planning to study more species such as the Barbary deer and evaluate the role of other possible hosts and their effect on the prevalence of tick-borne pathogens.

* Materials and Methods

- The materials and methods presented details of the overall studies.
- However, it would be better to abbreviate the study site and the tick sampling part.

Thank you for this comment. The study site information (line 123) and tick sampling (line 160) have been summarized.

- Please describe how ticks collected from animals were handled.

Collected ticks from vegetation and animals were handled in the same manner. As not to repeat the same information three times in the Tick sampling description, a final sentence was added (line 186-188) describing the method employed for all collected ticks:

“Ticks collected from vegetation, wild rabbits and addax were placed into plastic vials containing RNAlater (Thermo Fisher, Waltham, MA, USA) and stored at -20 ºC until further analysis.”

* Results

- Line 234: Why did you indicate as "spp."?

When doing morphological identification of questing ticks using a stereomicroscope, we were not able to determine with certainty the *Rhipicephalus* tick’s species. Nevertheless, to solve this issue, the tick's DNA was extracted, and its molecular identification was performed, allowing its classification as *Rhipicephalus* *pusillus* (mentioned in line 321)*.*

* Discussion

- The discussion is qualified in terms of evaluating the results. However, the discussion is limited to evaluating the results. It would be better to compare with similar studies conducted worldwide using the same or similar techniques, which would enrich the text. It is recommended to include an evaluation of the results of recent similar studies conducted around the world in the discussion.

Thank you for this comment, in the discussion it has been added additional information discussing tick-borne rickettsiosis and *Coxiella burnetii* in Africa and Mediterranean areas. The following sentences have been added to the manuscript:

“Studies conducted in other regions of North Africa point to a prevalence of tick-borne rickettsiosis between 0.5 - 64%, depending on the *Rickettsia* species [20]” (lines 449-451)

“We detected a lower prevalence of *C. burnetii* (22.6% of questing ticks and 28.6% of addax ticks), compared to previous studies, which describe higher prevalences in the Mediterranean basin [16,53] or African countries [20]. Such differences may be attributed to different tick characteristics, the multitude of tick species and host communities present in both regions, which could condition the maintenance and/or increase the prevalence of the pathogen [1].” (lines 458-464).

**Reviewer #2:**

Ticks and tick-borne pathogens pose a serious threat worldwide. The study offers important findings that will help protect both human and wildlife health. I have some edits and comments in the attached pdf file for the authors to consider.

Thank you very much for your comments. Edits and suggestions have all been accepted. Comments have been addressed in the following matter.

- Line 187: Collected from Addax?

Sorry for not having been clear in the initial manuscript. Due to budget constrains only ticks from vegetation and rabbit samples were sequenced. Morphologically, most collected ticks from addax and vegetation were identified as *Hyalomma lusitanicum*, hence we only confirm the identification with vegetation ticks. The sentence was changed for: “Ticks used for molecular identification included four individual adult ticks collected from vegetation, confirming also the identification of addax’s ticks, and ticks collected from rabbits - four individual adult ticks, and three pools of nymphs, constituted by two to four nymphs.” (See lines 207-210)

- Line 236: Looks like the one nymph was excluded. Confirm and change this to 1230

Thank you for noticing this mistake. The number of ticks has been updated to 1230.

-Line 239: The numbers add up to 3753. It was previously stated that questing ticks were 3754. Check and revise.

Thank you once again for pointing out the error. There was a tick missing in zone D. The sentence has been modified to: “Spatially, more questing ticks were collected in zones C and A (1948 and 1127, respectively) than in B and D (631 and 48, respectively).” (see lines 263-264).

- Line 288: You used the molecular method to confirm the species of the adult and nymph ticks. However, the larvae were not mentioned. Did you use the molecular method to confirm the tick species that were larvae? Larvae are usually difficult to identify using morphology.

Molecular identification was only performed in adult and nymph species. There was a mistake in the classification of larvae ticks collected from rabbits, as larvae ticks were only identified up to the genus level. This issue has been corrected as: “Ticks were morphologically classified as *H. lusitanicum* (4 adults), *Hyalomma* spp. (347 nymphs and larvae) and *Rhipicephalus pusillus* (583 adults, 185 nymphs), and *Rhipicephalus* spp. (18 larvae).” (see line 280).

- Line 329: This sentence is wordy. Can it be rephrased?

Thank you for this comment. The sentence was rewritten to: “The *16S rDNA* PCR-positive sample (Isolate 23) to *Rickettsia* spp. shared 100% nucleotide identity with *R. aeschlimannii* retrieved in Spain from *H. rufipes* and *H. marginatum* ticks (GenBank accession numbers MW394393 and MW394392, respectively) (Fig 6).” (see lines 355-359).

- Line 407: Was a thorough search done to confirm this is the first worldwide report? If not, its best you limit it to North Africa or Africa.

Thank you for this comment. A thorough search was not performed, and although no articles were found on *Rickettsia aeschlimannii* in *Hyalomma lusitanicum* questing ticks during the bibliographic search, we are not able to confirm this is the first detection worldwide. Hence, the sentence has been changed to: “To our knowledge, we report the first detection of *R. aeschlimannii* in *H. lusitanicum* questing ticks in Africa, along with the identification of *C. burnetii*.” (see lines 442-444).

**Reviewer #3:**

The study focuses on a timely and highly relevant topic: the biodiversity of ticks in a natural Mediterranean area, their seasonal ecology, the interactions between ticks and wild hosts, and the presence of important zoonotic pathogens. The significance of the research is high, considering the role of ticks in disease transmission and the potential impact of climate change on the dynamics of arthropod vector populations. The work is generally well-structured: the introduction, methods, and results are adequately described. The discussion effectively contextualizes the findings within the ecological and health framework of the region, providing a valuable overview of the importance of seasonal and environmental dynamics in the distribution of ticks and pathogens.

References to the existing literature are appropriate and contribute to reinforcing hypotheses regarding host-vector interactions. The focus on climate change and its possible consequences on tick distribution is a strong point, aligning with the growing concern about emerging zoonotic risks. The study’s limitations are acknowledged, particularly regarding the limited sampling timeframe and the lack of pathogen analyses in rabbits. The emphasis on a One Health approach is rightly highlighted as necessary to address the complexity of the issue.

Thank you very much for your comment. It is highly appreciated.

**Response to the editors’ concerns:**

Thank you very much for the comments and improvements on the manuscript. All comments have been addressed, and a detail description is presented on each comment. An additional sentence has been included in the acknowledgements, that we would appreciate if it would be included in the final version (lines 506-510).

Manuscript needs language editing as some words are spelled wrong and some phrases lack proper English language construction "It's a must"

The manuscript has been revised to correct its English and misspelled words.

Write Species names in the phylogenetic tree in italics "It's a must"

Thank you for this comment, this has been emended.

Figures are not clear and need to be resubmitted after resolution adjustment "It's a must"

We agree, figures have been corrected according to specified comments. Species names have been corrected to italic and figures resolution has been improved.

The whole manuscript should be shortened especially the materials and Methods section which contains many Introduction sentences

According to reviewers' comments, we have shorted the manuscript.

These are some edits also

'Corresponding Authors' is written 'Autors'

Misspelled words have been addressed.

1. Please ensure that your manuscript meets PLOS ONE's style requirements, including those for file naming. The PLOS ONE style templates can be found at

<https://journals.plos.org/plosone/s/file?id=wjVg/PLOSOne_formatting_sample_main_body.pdf> and [https://journals.plos.org/plosone/s/file?id=ba62/PLOSOne_formatting_sample_title_authors_affiliations.pdf](https://track.editorialmanager.com/CL0/https:%2F%2Fjournals.plos.org%2Fplosone%2Fs%2Ffile%3Fid=ba62%2FPLOSOne_formatting_sample_title_authors_affiliations.pdf/1/010f019730b4790b-2448cc0f-a397-43e5-9c5d-6b783ba63a95-000000/W8jCbIefHggnZVTABsm5_Gk2yBZApzethiZ1a6sK90U=213)

Manuscript and file name style have been modified according to PLOS ONE requirements.

2. In your Methods section, please provide additional information regarding the permits you obtained for the work. Please ensure you have included the full name of the authority that approved the field site access and, if no permits were required, a brief statement explaining why.

It has been included a paragraph in the methodology addressing this comment in lines 189-192 as: “Ticks’ collection from vegetation, as well as from dead and live animals, was carried out as part of a private initiative for monitoring threatened species in the Maamora Forest, following approved ethical protocols for wildlife capture and management (no permits were required).” The protocols employed followed international standards.

3. We noticed you have some minor occurrence of overlapping text with the following previous publication(s), which needs to be addressed:

<https://www.sciencedirect.com/science/article/pii/S2352771423000678?via%3Dihub>

<https://pubmed.ncbi.nlm.nih.gov/37973690/>

<https://link.springer.com/article/10.1007/s10493-023-00863-7>

In your revision ensure you cite all your sources (including your own works), and quote or rephrase any duplicated text outside the methods section. Further consideration is dependent on these concerns being addressed.

Thank you for this comment, this issue has been addressed by rephasing sentences and including the reference of mentioned articles.

4. Thank you for stating in your Funding Statement: [This research was partly supported by the 2022-GRIN-34227 grant, funded by the University of Castile-La Mancha (UCLM), Spain and EU-FEDER. R. Vaz-Rodrigues was supported by a doctoral contract (2022-PRED-20675), from UCLM and co-financed by the European Social Fund (ESF).].

Please provide an amended statement that declares *all* the funding or sources of support (whether external or internal to your organization) received during this study, as detailed online in our guide for authors at <http://journals.plos.org/plosone/s/submit-now>.  Please also include the statement “There was no additional external funding received for this study.” in your updated Funding Statement.

Please include your amended Funding Statement within your cover letter. We will change the online submission form on your behalf.

An amendment of the Funding Statement has been included in the manuscript as, “This research was supported by the 2022-GRIN-34227 grant, funded by the University of Castile-La Mancha (UCLM), Spain and EU-FEDER. R. Vaz-Rodrigues was supported by a doctoral contract (2022-PRED-20675), from UCLM and co-financed by the European Social Fund (ESF). There was no additional external funding received for this study.”

5. Please include captions for your Supporting Information files at the end of your manuscript, and update any in-text citations to match accordingly. Please see our Supporting Information guidelines for more information: [http://journals.plos.org/plosone/s/supporting-information](http://track.editorialmanager.com/CL0/http:%2F%2Fjournals.plos.org%2Fplosone%2Fs%2Fsupporting-information/1/010f019730b4790b-2448cc0f-a397-43e5-9c5d-6b783ba63a95-000000/6kl8iB_0OLQC-va-0xvb42-VilHyYzh2Amz7mLoBWNc=213).

Captions of supporting material have been included after the references.

6. Please review your reference list to ensure that it is complete and correct. If you have cited papers that have been retracted, please include the rationale for doing so in the manuscript text, or remove these references and replace them with relevant current references. Any changes to the reference list should be mentioned in the rebuttal letter that accompanies your revised manuscript. If you need to cite a retracted article, indicate the article’s retracted status in the References list and also include a citation and full reference for the retraction notice.

References have been actualized according to retracted or additional text, following the reviewers’ comments. Previous articles cited with the numbers 25 and 26 have been retracted. Eliminated references include the articles:

25. Segura A, Rotger A, Rodríguez-Caro RC. Hidden Threats to Persistence: Changes in Population Structure Can Affect Well-Preserved Spur-Thighed Tortoise Populations. Herpetologica. 2025;81: 23–33. doi:10.1655/Herpetologica-D-23-00066
26. Filahi S, Tanarhte M, Mouhir L, El Morhit M, Tramblay Y. Trends in indices of daily temperature and precipitations extremes in Morocco. Theor Appl Climatol. 2016;124: 959–972. doi:10.1007/s00704-015-1472-4

In addition, in the revised manuscript, reference 53 has been added:

53. González J, González MG, Valcárcel F, Sánchez M, Martín-Hernández R, Tercero JM, et al. Prevalence of Coxiella burnetii (Legionellales: Coxiellaceae) Infection Among Wildlife Species and the Tick Hyalomma lusitanicum (Acari: Ixodidae) in a Meso-Mediterranean Ecosystem. J Med Entomol. 2020;57: 551–556. doi:10.1093/jme/tjz169

The final list of refences is as follows:

1. de la Fuente J, Contreras M, Estrada-Peña A, Cabezas-Cruz A. Targeting a global health problem: Vaccine design and challenges for the control of tick-borne diseases. Vaccine. 2017;35: 5089–5094. doi:10.1016/j.vaccine.2017.07.097
2. Estrada-Peña A, Mihalca AD, Petney TN. Ticks of Europe and North Africa: A Guide to Species Identification. Springer; 2017.
3. Sonenshine DE, Roe RM. Biology of Ticks Volume 1. OUP USA; 2013.
4. Allan BF, Dutra HP, Goessling LS, Barnett K, Chase JM, Marquis RJ, et al. Invasive honeysuckle eradication reduces tick-borne disease risk by altering host dynamics. Proc Natl Acad Sci U S A. 2010;107: 18523–18527. doi:10.1073/pnas.1008362107
5. Valcárcel F, Elhachimi L, Vilá M, Tomassone L, Sánchez M, Selles SMA, et al. Emerging Hyalomma lusitanicum: From identification to vectorial role and integrated control. Med Vet Entomol. 2023;37: 425–459. doi:10.1111/mve.12660
6. Daniel M, Cerný V, Dusbábek F, Honzáková E, Olejnícek J. Influence of microclimate on the life cycle of the common tick Ixodes ricinus (L.) in thermophilic oak forest. Folia Parasitol (Praha). 1976;23: 327–342.
7. Medlock JM, Hansford KM, Bormane A, Derdakova M, Estrada-Peña A, George J-C, et al. Driving forces for changes in geographical distribution of Ixodes ricinus ticks in Europe. Parasit Vectors. 2013;6: 1. doi:10.1186/1756-3305-6-1
8. Yessinou RE, Koumassou A, Galadima HB, Nanoukon-Ahigan H, Farougou S, Pfeffer M. Tick Diversity and Distribution of Pathogen in Ticks Collected from Wild Animals and Vegetation in Africa. Pathogens. 2025;14: 116. doi:10.3390/pathogens14020116
9. Segura A, Rodríguez O, Ruiz-Fons F, Acevedo P. Tick parasitism in the Mediterranean spur-thighed tortoise in the Maamora forest, Morocco. Ticks Tick-Borne Dis. 2019;10: 286–289. doi:10.1016/j.ttbdis.2018.11.002
10. Norte AC, Harris DJ, Silveira D, Nunes CS, Núncio MS, Martínez EG, et al. Diversity of microorganisms in Hyalomma aegyptium collected from spur-thighed tortoise (Testudo graeca) in North Africa and Anatolia. Transbound Emerg Dis. 2022;69: 1951–1962. doi:10.1111/tbed.14188
11. Segura A, Rafael M, Vaz-Rodrigues R, Rodríguez O, Gortázar C, de la Fuente J. Tick infestation in spur-thighed tortoise population: a pilot study for unraveling epidemiological patterns and demographic consequences. Exp Appl Acarol. 2023 [cited 21 Nov 2023]. doi:10.1007/s10493-023-00863-7
12. Keysary A, Eremeeva ME, Leitner M, Din AB, Wikswo ME, Mumcuoglu KY, et al. Spotted Fever Group Rickettsiae in Ticks Collected from Wild Animals in Israel. Am J Trop Med Hyg. 2011;85: 919–923. doi:10.4269/ajtmh.2011.10-0623
13. Said Y, Lahmar S, Dhibi M, Rjeibi MR, Jdidi M, Gharbi M. First survey of ticks, tick-borne pathogens (Theileria, Babesia, Anaplasma and Ehrlichia) and Trypanosoma evansi in protected areas for threatened wild ruminants in Tunisia. Parasitol Int. 2021;81: 102275. doi:10.1016/j.parint.2020.102275
14. Rodríguez O, de la Fuente G, Fernández de Mera IG, Vaz-Rodrigues R, Gortázar C, de la Fuente J. The Saharan antelope addax (Addax nasomaculatus) as a host for Hyalomma marginatum, tick vector of Crimean-Congo hemorrhagic fever virus. Ticks Tick-Borne Dis. 2022;13: 102034. doi:10.1016/j.ttbdis.2022.102034
15. Loftis AD, Reeves WK, Szumlas DE, Abbassy MM, Helmy IM, Moriarity JR, et al. Population Survey of Egyptian Arthropods for Rickettsial Agents. Ann N Y Acad Sci. 2006;1078: 364–367. doi:10.1196/annals.1374.072
16. Sánchez M, Valcárcel F, González J, González MG, Martín-Hernández R, Tercero JM, et al. Seasonality of Coxiella burnetii among Wild Rabbits (Oryctolagus cuniculus) and the Hyalomma lusitanicum (Acari: Ixodidae) in a Meso-Mediterranean Ecosystem. Pathogens. 2022;11: 36. doi:10.3390/pathogens11010036
17. Ruiz-Fons F, Fernández-de-Mera IG, Acevedo P, Gortázar C, de la Fuente J. Factors Driving the Abundance of Ixodes ricinus Ticks and the Prevalence of Zoonotic I. ricinus-Borne Pathogens in Natural Foci. Appl Environ Microbiol. 2012;78: 2669–2676. doi:10.1128/AEM.06564-11
18. Wikel SK. Ticks and Tick-Borne Infections: Complex Ecology, Agents, and Host Interactions. Vet Sci. 2018;5: 60. doi:10.3390/vetsci5020060
19. d’Oliveira C, van der Weide M, Jacquiet P, Jongejan F. Detection of Theileria annulata by the PCR in ticks (Acari: Ixodidae) collected from cattle in Mauritania. Exp Appl Acarol. 1997;21: 279–291. doi:10.1023/A:1018455223462
20. Abdelbaset AE, Kwak ML, Nonaka N, Nakao R. Human-biting ticks and zoonotic tick-borne pathogens in North Africa: diversity, distribution, and trans-Mediterranean public health challenges. One Health Amst Neth. 2023;16: 100547. doi:10.1016/j.onehlt.2023.100547
21. Vatansever Z, Gargili A, Aysul NS, Sengoz G, Estrada-Peña A. Ticks biting humans in the urban area of Istanbul. Parasitol Res. 2008;102: 551–553. doi:10.1007/s00436-007-0809-z
22. Bursali A, Tekin S, Keskin A, Ekici M, Dundar E. Species diversity of ixodid ticks feeding on humans in Amasya, Turkey: seasonal abundance and presence of Crimean-Congo hemorrhagic fever virus. J Med Entomol. 2011;48: 85–93. doi:10.1603/me10034
23. Fennane M, Rejdali M. The world largest cork oak Maamora forest: challenges and the way ahead. Flora Mediterr. 2015;25: 277–285. doi:10.7320/FlMedit25SI.277
24. IUCN. IUCN SSC Antelope Specialist Group. Addax nasomaculatus. The IUCN Red List of Threatened Species 2016: e.T512A50180603. IUCN. 2016 [cited 29 Nov 2023]. doi:10.2305/IUCN.UK.2016-2.RLTS.T512A50180603.en
25. Said L, Assmaa A, Najib G, Gmira N. Contribution à l’évaluation de la pression pastorale dans la forêt de la Maamora. Parcours forestiers et surpâturage. Nat Tecnol. 2014; 39–50.
26. Coimbra-Dores MJ, Maia-Silva M, Marques W, Oliveira AC, Rosa F, Dias D. Phylogenetic insights on Mediterranean and Afrotropical Rhipicephalus species (Acari: Ixodida) based on mitochondrial DNA. Exp Appl Acarol. 2018;75: 107–128. doi:10.1007/s10493-018-0254-y
27. Moraga Fernández A, Ortiz JA, Jabbar A, Ghafar A, Cabezas-Cruz A, de la Fuente G, et al. Fatal cases of bovine anaplasmosis in a herd infected with different Anaplasma marginale genotypes in southern Spain. Ticks Tick Borne Dis. 2022; 101864–101864.
28. Rjeibi MR, Amairia S, Mhadhbi M, Rekik M, Gharbi M. Detection and molecular identification of Anaplasma phagocytophilum and Babesia spp. infections in Hyalomma aegyptium ticks in Tunisia. Arch Microbiol. 2022;204: 385. doi:10.1007/s00203-022-02995-7
29. Moraga-Fernández A, Ruiz-Fons F, Habela MA, Royo-Hernández L, Calero-Bernal R, Gortazar C, et al. Detection of new Crimean-Congo haemorrhagic fever virus genotypes in ticks feeding on deer and wild boar, Spain. Transbound Emerg Dis. 2021;68: 993–1000. doi:10.1111/tbed.13756
30. Moraga-Fernández A, Chaligiannis Ι, Cabezas-Cruz A, Papa A, Sotiraki S, de la Fuente J, et al. Molecular identification of spotted fever group Rickettsia in ticks collected from dogs and small ruminants in Greece. Exp Appl Acarol. 2019;78: 421–430. doi:10.1007/s10493-019-00392-2
31. Fernández de Mera IG, Zivkovic Z, Bolaños M, Carranza C, Pérez-Arellano JL, Gutiérrez C, et al. Rickettsia massiliae in the Canary Islands. Emerg Infect Dis. 2009;15: 1869–1870. doi:10.3201/eid1511.090681
32. Roux V, Rydkγna E, Eremeeva M, Raoult D. Citrate Synthase Gene Comparison, a New Tool for Phylogenetic Analysis, and Its Application for the Rickettsiae†. Int J Syst Evol Microbiol. 1997;47: 252–261. doi:10.1099/00207713-47-2-252
33. Bonsergent C, Vittecoq M, Leray C, Burkart L, McCoy KD, Malandrin L. Characterization and diversity of Babesia sp. YLG, a new member of the Peircei group infecting Mediterranean yellow-legged gulls (Larus michahellis). Ticks Tick-Borne Dis. 2022;13: 101852. doi:10.1016/j.ttbdis.2021.101852
34. Akaike H. A new look at the statistical model identification. IEEE Trans Autom Control. 1974;19: 716–723. doi:10.1109/TAC.1974.1100705
35. R Core Team. R: The R Project for Statistical Computing. Vienna, Austria: R Foundation for Statistical Computing; 2023. Available: <https://www.R-project.org/>
36. Estrada-Peña A, Bouattour A, J-L C, Walker A. Ticks of Domestic Animals in Africa. A Guide to Identification of Species. 2004.
37. Castro-Scholten S, Caballero-Gómez J, Martínez R, Nadales-Martín BJ, Cano-Terriza D, Jiménez-Martín D, et al. Occurrence of Coxiella burnetii in wild lagomorphs and their ticks in Spanish Mediterranean ecosystems. Zoonoses Public Health. 2024;71: 549–559. doi:10.1111/zph.13155
38. Peralbo-Moreno A, Baz-Flores S, Cuadrado-Matías R, Barroso P, Triguero-Ocaña R, Jiménez-Ruiz S, et al. Environmental factors driving fine-scale ixodid tick abundance patterns. Sci Total Environ. 2022;853: 158633. doi:10.1016/j.scitotenv.2022.158633
39. Estrada-Peña A, Venzal JM. Climate niches of tick species in the Mediterranean region: modeling of occurrence data, distributional constraints, and impact of climate change. J Med Entomol. 2007;44: 1130–1138. doi:10.1603/0022-2585(2007)44[1130:cnotsi]2.0.co;2
40. Ogden NH, Ben Beard C, Ginsberg HS, Tsao JI. Possible Effects of Climate Change on Ixodid Ticks and the Pathogens They Transmit: Predictions and Observations. J Med Entomol. 2021;58: 1536–1545. doi:10.1093/jme/tjaa220
41. Keesing F, Ostfeld RS, Young TP, Allan BF. Cattle and rainfall affect tick abundance in central Kenya. Parasitology. 2018;145: 345–354. doi:10.1017/S003118201700155X
42. Bugmyrin SV, Bespyatova LA, Korotkov YuS. Long-term dynamics of Ixodes persulcatus (Acari: Ixodidae) abundance in the north–west of its range (Karelia, Russia). Exp Appl Acarol. 2019;77: 229–240. doi:10.1007/s10493-019-00342-y
43. Intergovernmental Panel on Climate Change (IPCC). Climate Change 2013 – The Physical Science Basis: Working Group I Contribution to the Fifth Assessment Report of the Intergovernmental Panel on Climate Change. Cambridge: Cambridge University Press; 2014. doi:10.1017/CBO9781107415324
44. Polade SD, Gershunov A, Cayan DR, Dettinger MD, Pierce DW. Precipitation in a warming world: Assessing projected hydro-climate changes in California and other Mediterranean climate regions. Sci Rep. 2017;7: 10783. doi:10.1038/s41598-017-11285-y
45. Driouech F, Stafi H, Khouakhi A, Moutia S, Badi W, ElRhaz K, et al. Recent observed country-wide climate trends in Morocco. Int J Climatol. 2021;41: E855–E874. doi:10.1002/joc.6734
46. Gray JS, Dautel H, Estrada-Peña A, Kahl O, Lindgren E. Effects of climate change on ticks and tick-borne diseases in Europe. Interdiscip Perspect Infect Dis. 2009;2009: 593232. doi:10.1155/2009/593232
47. Buczek AM, Buczek W, Buczek A, Bartosik K. The Potential Role of Migratory Birds in the Rapid Spread of Ticks and Tick-Borne Pathogens in the Changing Climatic and Environmental Conditions in Europe. Int J Environ Res Public Health. 2020;17: 2117. doi:10.3390/ijerph17062117
48. Raoult D, Fournier P-E, Abboud P, Caron F. First Documented Human Rickettsia aeschlimannii Infection. Emerg Infect Dis. 2002;8: 748–749. doi:10.3201/eid0807.010480
49. Germanakis A, Chochlakis D, Angelakis E, Tselentis Y, Psaroulaki A. Rickettsia aeschlimannii infection in a Man, Greece. Emerg Infect Dis. 2013;19. doi:10.3201/eid1907.130232
50. Chisu V, Leulmi H, Masala G, Piredda M, Foxi C, Parola P. Detection of Rickettsia hoogstraalii, Rickettsia helvetica, Rickettsia massiliae, Rickettsia slovaca and Rickettsia aeschlimannii in ticks from Sardinia, Italy. Ticks Tick-Borne Dis. 2017;8: 347–352. doi:10.1016/j.ttbdis.2016.12.007
51. Chochlakis D, Ioannou I, Sandalakis V, Dimitriou T, Kassinis N, Papadopoulos B, et al. Spotted Fever Group Rickettsiae in Ticks in Cyprus. Microb Ecol. 2012;63: 314–323. doi:10.1007/s00248-011-9926-4
52. Maurin M, Raoult D. Q Fever. Clin Microbiol Rev. 1999;12: 518–553. doi:10.1128/cmr.12.4.518
53. González J, González MG, Valcárcel F, Sánchez M, Martín-Hernández R, Tercero JM, et al. Prevalence of Coxiella burnetii (Legionellales: Coxiellaceae) Infection Among Wildlife Species and the Tick Hyalomma lusitanicum (Acari: Ixodidae) in a Meso-Mediterranean Ecosystem. J Med Entomol. 2020;57: 551–556. doi:10.1093/jme/tjz169
54. Eldin C, Mélenotte C, Mediannikov O, Ghigo E, Million M, Edouard S, et al. From Q Fever to Coxiella burnetii Infection: a Paradigm Change. Clin Microbiol Rev. 2016;30: 115–190. doi:10.1128/cmr.00045-16
55. Vanderburg S, Rubach MP, Halliday JEB, Cleaveland S, Reddy EA, Crump JA. Epidemiology of Coxiella burnetii Infection in Africa: A OneHealth Systematic Review. PLoS Negl Trop Dis. 2014;8: e2787. doi:10.1371/journal.pntd.0002787
56. González-Barrio D, Almería S, Caro MR, Salinas J, Ortiz JA, Gortázar C, et al. Coxiella burnetii Shedding by Farmed Red Deer (Cervus elaphus). Transbound Emerg Dis. 2015;62: 572–574. doi:10.1111/tbed.12179
57. Yang S, Rothman RE. PCR-based diagnostics for infectious diseases: uses, limitations, and future applications in acute-care settings. Lancet Infect Dis. 2004;4: 337–348. doi:10.1016/S1473-3099(04)01044-8
58. Kleinerman G, Baneth G, Mumcuoglu KY, van Straten M, Berlin D, Apanaskevich DA, et al. Molecular Detection of Rickettsia africae, Rickettsia aeschlimannii, and Rickettsia sibirica mongolitimonae in Camels and Hyalomma spp. ticks from Israel. Vector-Borne Zoonotic Dis. 2013;13: 851–856. doi:10.1089/vbz.2013.1330
59. Abdelkadir K, Palomar AM, Portillo A, Oteo JA, Ait-Oudhia K, Khelef D. Presence of Rickettsia aeschlimannii, ‘Candidatus Rickettsia barbariae’ and Coxiella burnetii in ticks from livestock in Northwestern Algeria. Ticks Tick-Borne Dis. 2019;10: 924–928. doi:10.1016/j.ttbdis.2019.04.018
